# Placenta-derived proteins across gestation in healthy pregnancies—a novel approach to assess placental function?

**DOI:** 10.1186/s12916-022-02415-z

**Published:** 2022-07-01

**Authors:** Maren-Helene Langeland Degnes, Ane Cecilie Westerberg, Manuela Zucknick, Theresa L. Powell, Thomas Jansson, Tore Henriksen, Marie Cecilie Paasche Roland, Trond Melbye Michelsen

**Affiliations:** 1 Department of Obstetrics, Division of Obstetrics and Gynecology, Oslo University Hospital Rikshospitalet, Oslo, Norway; 2 Department of Biostatistics, Oslo Centre for Biostatistics and Epidemiology, University of Oslo, Oslo, Norway; 3 Division of Reproductive Sciences, Department of Obstetrics and Gynecology, University of Colorado Anschutz Medical Campus, Aurora, CO USA; 4 Section of Neonatology, Department of Pediatrics, University of Colorado Anschutz Medical Campus, Aurora, CO USA; 5 Institute of Clinical Medicine, Faculty of Medicine, University of Oslo, Oslo, Norway; 6 National Research Centre for Women’s Health, Oslo University Hospital, Rikshospitalet, Oslo, Norway

**Keywords:** Aptamer, Biomarkers, Four vessel sampling, Human, Placenta, Pregnancy, Proteomics, SomaLogic

## Abstract

**Background:**

Placenta-derived proteins in the systemic maternal circulation are suggested as potential biomarkers for placental function. However, the identity and longitudinal patterns of such proteins are largely unknown due to the inaccessibility of the human placenta and limitations in assay technologies. We aimed to identify proteins derived from and taken up by the placenta in the maternal circulation. Furthermore, we aimed to describe the longitudinal patterns across gestation of placenta-derived proteins as well as identify placenta-derived proteins that can serve as reference curves for placental function.

**Methods:**

We analyzed proteins in plasma samples collected in two cohorts using the Somalogic 5000-plex platform. Antecubital vein samples were collected at three time points (gestational weeks 14–16, 22–24, and 30–32) across gestation in 70 healthy pregnancies in the longitudinal STORK cohort. In the cross sectional 4-vessel cohort, blood samples were collected simultaneously from the maternal antecubital vein (AV), radial artery (RA), and uterine vein (UV) during cesarean section in 75 healthy pregnancies. Placenta-derived proteins and proteins taken up by the placenta were identified using venoarterial differences (UV-RA). Placenta-derived proteins were defined as placenta-specific by comparison to the venoarterial difference in the antecubital vein-radial artery (AV-RA). These proteins were described longitudinally based on the STORK cohort samples using a linear mixed effects model per protein. Using a machine learning algorithm, we identified placenta-derived proteins that could predict gestational age, meaning that they closely tracked gestation, and were potential read-outs of placental function.

**Results:**

Among the nearly 5000 measured proteins, we identified 256 placenta-derived proteins and 101 proteins taken up by the placenta (FDR < 0.05). Among the 256 placenta-derived proteins released to maternal circulation, 101 proteins were defined as placenta-specific. These proteins formed two clusters with distinct developmental patterns across gestation. We identified five placenta-derived proteins that closely tracked gestational age when measured in the systemic maternal circulation, termed a “placental proteomic clock.”

**Conclusions:**

Together, these data may serve as a first step towards a reference for the healthy placenta-derived proteome that can be measured in the systemic maternal circulation and potentially serve as biomarkers of placental function. The “placental proteomic clock” represents a novel concept that warrants further investigation. Deviations in the proteomic pattern across gestation of such proteomic clock proteins may serve as an indication of placental dysfunction.

**Supplementary Information:**

The online version contains supplementary material available at 10.1186/s12916-022-02415-z.

## Background

A well-functioning, healthy placenta is necessary for a successful pregnancy. In healthy pregnancies, the placenta produces and releases hormones and other signaling molecules that have major impacts on maternal physiology by promoting the endocrine, metabolic, cardiovascular, and immune changes that occur as an adaptation to pregnancy. A dysfunctional placenta, which is evident in several of the “great obstetrical syndromes” [1], have altered release of substances due to hypoxia, oxidative stress, or inflammation [2–4]. This may affect the circulating levels of substances, including proteins, in the maternal systemic circulation.

Development of minimally invasive approaches to monitor placental function across gestation would represent a paradigm shift in terms of early diagnosis and monitoring of placenta-related pregnancy complications, as well as provide critical knowledge necessary to develop intervention strategies targeting placental function. Efforts to test clinical relevance of biochemical markers of placental function have not been successful thus far, and such studies have only included a very limited number of placenta-derived proteins (such as human placental lactogen (hPL), placental growth factor (PGF), and pregnancy-associated plasma protein A (PAPP-A)) [5, 6]. Thus, no biomarker for placental function is universally accepted and implemented in the clinic. Aghaeepour et al. suggested the establishment of a proteomic clock of healthy pregnancy by use of proteins with precisely timed changes across gestation, so that pregnancies with deviations from this proteomic pattern could be identified as “at risk” [7]. However, whether the placenta was the origin of the identified “clock proteins” was unknown.

Placenta-derived biomarkers have been associated with disturbance in placental vascularization, metabolism, inflammation, and immunological responses observed in common pregnancy complications [8, 9]. For example, the placenta releases PGF and soluble Fms-like tyrosine kinase 1 (sFlt-1) [3]. PGF is important for normal angiogenesis by promoting proliferation, migration, and survival of endothelial cells through binding to the transmembrane receptors vascular endothelial growth factor receptor 1 (VEGFR 1) and VEGFR 2. The soluble VEGFR receptor, sFlt-1, can bind PGF and thus inhibit its angiogenic effects. Higher sFlt-1/PGF ratio is associated with increased risk of preeclampsia [3, 10, 11]. However, the identity and longitudinal pattern of all placenta-derived proteins in the maternal circulation has not been fully explored both due to the inaccessibility of the human placenta and technical challenges in measuring large numbers of proteins. Thus, it is plausible that currently unknown proteins may be identified and are relevant as biomarkers for placental function.

The current understanding of which proteins the human placenta releases into the maternal circulation is largely based on studies of human ex vivo perfusion studies, placental explants, isolated primary human trophoblasts, and immortalized trophoblast cell lines as well as indirect evidence showing transient increased levels of maternal proteins during pregnancy [12]. According to the Human Protein Atlas, transcriptome analysis shows that 65% (*n* = 13074) of all human proteins (*n* = 20090) are expressed in the placenta, and 288 of these genes show an elevated expression in the placenta compared to other tissue types [13, 14]. These data indicate that the placenta, like other tissues, relies on the expression of “housekeeping” genes to ensure basic cellular functions but that several genes are especially important in the placenta and probably some are “placenta-specific” as their expression is enriched. These findings are based on RNA sequencing of placental tissue samples (which is a mixture of cell types, trophoblasts, endothelial and immune cells) collected at birth. To identify placenta-derived proteins, we used the 4-vessel sampling method, allowing blood sample collection from the human placental circulation in vivo [15]. Blood samples are drawn from the incoming and outgoing vessels on the maternal (radial artery and uterine vein) side of the placenta while in situ during elective cesarean section, enabling calculation of differences across the maternal arterial and venous circulation.

We have previously published exploratory data from a pilot study (*n* = 35) of the 4-vessel cohort using a previous SomaLogic platform with 1310 protein specific aptamers [16]. This study indicated that 34 proteins were significantly released into the maternal circulation from the placenta. Further analysis of these 34 proteins in a small longitudinal cohort (*n* = 8, measurements at 3 time points across gestation) showed that the abundance (relative fluorescence units (RFUs)) of 8 out of 34 proteins changed significantly during gestation, including PGF which increased with advancing gestation in accordance with previous reports.

In this study, we included more participants, used a larger proteomic platform, the 5000-multiplex SomaScan, and expanded the scope of the analysis. First, we aimed to identify placenta-derived proteins and proteins taken up by the placenta on the maternal side using in vivo 4-vessel plasma samples from 75 healthy pregnancies. Second, we characterized longitudinal patterns of placenta-derived proteins in the maternal circulation across gestation using antecubital vein samples from 70 healthy pregnant women in the prospective, longitudinal STORK cohort. Last, we identified placenta-derived proteins in the maternal circulation that tightly track with gestational age: a “placental proteomic clock” of healthy pregnancy, inspired by the work of Aghaeepour et al. [7]. The latter involves a novel concept that may provide a basis to assess placental function through repeated blood samples and measurement of placenta-derived proteins across gestation.

## Methods

### Design and study population

#### Four vessel cohort

The 4-vessel cohort is a cross-sectional study of healthy, non-smoking pregnant women with uncomplicated singleton pregnancies scheduled for elective cesarean section at Oslo University Hospital, Rikshospitalet, between October 2012 and June 2016. Pre-labor cesarean delivery was performed under spinal anesthesia. We sampled from the maternal radial artery, antecubital vein, and uterine vein on the anterolateral surface of the uterus immediately before uterine incision. After delivery of the infant and before detachment of the placenta, we collected blood samples from the umbilical artery and vein. In this study, we focus only on maternal samples. All blood samples were immediately transferred to EDTA vacutainers, kept on ice, centrifuged within 30 min (6 °C, 2500g, 20 min), and stored at − 80 °C. The method has been described in detail elsewhere [15].

We applied Liu and Hwang’s “quick calculation for sample size” [17] and corresponding R package [18] to estimate the required sample size to achieve at least 80% statistical power for large-scale screening to identify proteins with non-zero log_2_ RFU maternal venoarterial differences (|UV - RA|) by paired *t*-tests, while adjusting for multiple testing by controlling the false discovery rate at a fixed level 0.1. We estimated the expected distributions of the |UV - RA| values from the pilot study [16] and found that a sample size of 75 achieves the required power, assuming that the proportion of differential proteins is at least 5%.

Consequently, proteomic analysis was performed on a subsample of 75 out of 179 recruited healthy women (based on complete collected data according to the study protocol: (i) plasma from 5 vessels, (ii) ultrasound measures on the maternal side of placenta, (iii) placental homogenate, (iv) fetal ultrasound data, and (v) newborn caliper data collected at birth, in ranked order) [19]. Among the 75 participants in the current study, 28 women were included in the pilot study since these women were among the participants with most complete data. Due to practical challenges and time constrains, some samples (from some of the vessels) were not collected (Additional file 1: Table S1).

#### STORK cohort

The prospective, longitudinal STORK cohort recruited 1031 healthy women at Oslo University Hospital, Rikshospitalet, with uncomplicated singleton pregnancies during the first trimester of pregnancy between 2002 and 2008. Details about inclusion and study design have previously been published [20]. Exclusion criteria were multiple pregnancies, known pre-gestational diabetes, and severe chronic disease (lung, cardiac, gastrointestinal or renal). Proteomic analysis was performed on antecubital vein plasma samples obtained at three time points across gestation (gestational weeks 12–18, 21–26, and 29–33) in a random subsample of 70 healthy women.

#### Protein quantification by SomaLogic

Four-thousand-nine-hundred-seventy nine (4979) unique SOMAmer reagents (aptamers) were used to quantify proteins in 4-vessel and STORK plasma samples with a microarray-based platform called SomaScan assay version 4.0. The SomaScan assay is widely employed in high impact human proteomics studies [21–23]. In brief, EDTA-plasma was mixed with aptamers, and only aptamer- and protein–complexes with specific bindings were left in the dilution after several elution steps. Aptamers from these complexes were then isolated and added to microarrays where they hybridize to specific probes. Hybridized probes give a fluorescence signal and the total fluorescence signal per aptamer-specific probe results in the relative fluorescence units per protein [24]. Calibrators were included so that the degree of fluorescence represented a relative quantitative reflection of protein concentration measured in relative fluorescent units (RFU). See Additional file 1: S1.1 for more details. Samples were randomly allocated across 10 plates, while ensuring that samples from one individual were analyzed on the same plate to minimize intra-comparison variation. To account for variability in different steps in the SomaScan process, SomaLogic performed standard data normalization procedures. However, we did not include normalization based on SomaLogic’s biological reference population (“Adaptive Normalization by Maximum Likelihood”) because our pregnant Scandinavian women are expected to deviate from the American general population. After excluding proteins that did not pass SomaLogic’s quality control, we used 4564 proteins in further analyses, of which 3008 were expressed in the placenta according to the Human Protein Atlas [14]. Median intra- and interassay coefficients of variation were ~5% [25], and assay sensitivity was comparable to typical immunoassays, with a median lower limit of detection in the femtomolar (10^−15^ moles per liter) range [22].

### Statistical analyses

#### Preprocessing and description of samples

Data processing and analyses where performed in R version 4.1.1 [26] and IBM SPSS Statistics version 26 [27]. All RFU values were log2 transformed to minimize any deviation from normal distribution. Extreme outlier proteins among STORK samples (proteins with mean abundance across the three time points above the 98th quantile) were subjected to winsorization, i.e., shifted so that the mean across the time points corresponded to the 98th quantile. In this way, the extremes were reduced without removing the dynamic changes in protein abundance pattern across gestation. One participant’s samples showed an un-typical distribution of RFU values across all proteins, and we therefore excluded these.

#### Identification of proteins released and taken up by placenta

**Fig. 1:**
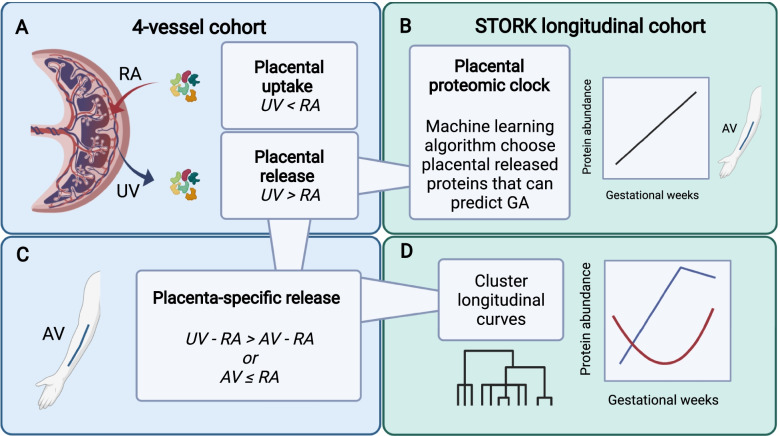
The four main aspects of our analysis (**A**–**D**). **A** Using paired *t*-tests, we compared levels/abundance (log2 RFUs) of proteins between the uterine vein (UV) and radial artery (RA) in 4-vessel samples to define placental uptake and release on the maternal side of the placenta. **B** A machine learning algorithm (the variable selector elastic net with stability selection) identified placenta-derived proteins that can predict gestational age, a “placental proteomic clock.” **C** We performed another set of comparisons to define placenta-specific released proteins (AV = antecubital vein). **D** Placenta-specific released proteins (based on **C**) were further characterized by the gestational changes in maternal protein levels (log2 RFU) using samples from the STORK cohort. Created with BioRender.com

To identify proteins as released or taken up by the placenta, we used paired *t*-tests between the uterine vein and radial artery (UV - RA) (Fig. 1A)^1^. A positive venoarterial log2 RFU difference on the maternal side of the placenta indicated placental release of proteins, whereas a negative venoarterial log2 RFU difference indicated uptake by the placenta (Table 1).

**Table 1:** Criteria for defining protein released or taken up by the placenta on maternal side

Definition of class	Type of statistical test per protein	Criteria per test
Placental release	Two-sided paired *t*-test for H_0_: *log*2(*RFU*_UV_) − *log* 2(*RFU*_RA_) = 0	• FDR-adjusted *p*-value < 0.05 • *t* statistic > 0
Placental uptake		• FDR-adjusted *p*-value < 0.05 • *t* statistic < 0

*UV* uterine vein, *RA* radial artery

Results were transformed from log2 RFU differences to percentage higher RFU in vein compared to artery for readability, see Equation 1. False discovery rate (FDR) was controlled by the method of Benjamini-Hochberg [28], and we inspected *p*-value histograms to diagnose experimental bias and false positives (Additional file 1: S1.2, and Fig. S1).

1 $$\left( mean\left({2}^{\mathit{\log}2\ RFU\ differences\ protein\ i}\right)-1\right)\ast 100=\% higher\ RFU\ in\ vein\ than\ artery$$

#### Median normalization of differences in protein abundance

There is evidence of a change in plasma water content from the uterine artery to the uterine vein due to a net movement of water from the mother to the fetus [29]. Thus, the protein concentration in the vessels with less water will be higher than vessels with more water. To account for possible biases between veins and arteries, mainly due to this “water shift,” we normalized the venoarterial differences across pairs of vessels per participant with median normalization^2^. Median normalization [30, 31] is based on the assumption that the majority of proteins are not taken up or released by the placenta, meaning that levels (log2 RFUs) of most proteins are similar in the arteries and the veins and that any bias affects all protein log2 RFU differences in each sample similarly.

#### Placenta-specific released proteins

Proteins with significantly higher positive venoarterial difference in the placenta as compared to the systemic circulation are defined as “placenta-specific released proteins” (Fig. 1C). The venoarterial difference in the arm was considered a proxy for any systemic venoarterial difference in the mother. To narrow in on proteins with specific relevance as placental proteins, we performed another set of *t*-tests comparing the venoarterial log2 RFU difference in the placenta (uterine vein-radial artery) with the arm (antecubital vein – radial artery) (see Additional file 1: S1.3 and Table S2 for detailed explanation). Proteins were defined as “placenta-specific released proteins” if they had statistically significant higher positive venoarterial log2 RFU difference in the placenta as compared to the arm or if the log2 RFU difference in the arm was negative or not significantly different from zero^3^.

#### Gene ontology enrichment analysis of placenta-specific released proteins

We performed gene ontology (GO) enrichment analysis using R-package *clusterProfiler* version 4.2.2 among the placenta-specific released proteins (101 proteins) [32]^4^. The enrichment analysis used was a competitive overrepresentation analysis performed on the list of placenta-specific released proteins against a background list including all proteins on the Somascan platform. We present results for human GO biological processes with FDR-adjusted *p*-value below 0.05 and a RichFactor above 0.15 to improve readability. RichFactor is the number of placenta-specific released proteins mapping to GO term/number of proteins measured by SOMAscan (background list) within the GO term.

#### Placenta-specific released proteins across gestation in the maternal circulation

Placenta-specific released proteins were described longitudinally based on antecubital vein samples collected in the prospective STORK cohort using linear mixed effects models per protein^3^. We modeled gestational age by cubic polynomial B-splines functions with the *splines* R package [26], and we included these functions as fixed effects in the model together with a fixed intercept. In addition, we included a random intercept for each participant. The mixed models predicted a mean curve per protein and we clustered these mean curves using hierarchical clustering with complete linkage by Pearson correlations (Fig. 1D). The number of clusters were determined using silhouette analysis through the *cluster* R-package version 2.1.0 [33].

#### Placental proteomic clock

To narrow in on placenta-derived proteins in Fig. 1A that could predict gestational age at sampling time point in the longitudinal STORK cohort, we used a machine learning algorithm (the variable selector; elastic net with stability selection (ENSS) through R-package *c060* 0.2-8 [34]); see Fig. 1B^5^. Two thirds of participant samples were defined as training cohort and the rest were validation cohort (1/3), and the clinical variables age, BMI, and nulliparity were included in the elastic net models as mandatory variables. Due to the dependence between repeated measurements, we used leave-one-participant-out cross validation to obtain prediction performance of a linear model including the proteins selected by the ENSS in the training cohort. The linear model resulting from the selected proteins was defined as a “placental proteomic clock.”

## Results

### Demographics

Seventy-five healthy women from the 4-vessel cohort and 70 healthy women from the STORK cohort participated (Table 2). The 4-vessel cohort participants had a mean (standard deviation, SD) age of 36.1 (3.8) years and 87.1% had higher education (> 15 years), whereas the STORK women had a mean (SD) age of 32.1 (3.6) years and 82.9% had higher education.

**Table 2:** Demographic and clinical characteristics of the 4-vessel and the STORK cohort

	4-vessel (***n*** = 75)					STORK (***n*** = 70)				
	*n*	%	Mean (SD)	Min	Max	*n*	%	Mean (SD)	Min	Max
**Mothers**										
Age (years)			36.0 (3.8)	28.0	44.0			32.1 (3.6)	24	41
Pre-pregnancy BMI^a^ (kg/m^2^)			23.7 (4.8)^e^	17.0	47.6			24.1 (2.9)	18.5	31.1
Systolic blood pressure (mmHg)^b^			109.9 (10.7)	90	135			110.0 (9.7)	90	130
Diastolic blood pressure (mmHg)^b^			68.1 (8.6)	48.0	83.0			66.3 (8.1)	45	80
No smoking during pregnancy^c^	71	94.7				69	98.5			
Higher education^d^	66	88.0				58	82.9			
Married/partner	71	94.7				70	100.0			
Nulliparity	17	22.7				35	50.0			
GA, 1st sampling								15.7 (1.2)^e^	12.1	18.1
GA, 2nd sampling								23.4 (1.0)	21.6	26.4
GA, 3rd sampling								31.3 (1.0)	29.4	33.6
**Infants**										
Sex (boys)	43	57.3				40	57.0			
GA at birth			38.8 (0.6)	37.0	41.0			40.3 (1.2)	37.6	42.6
Placental weight (g)			617.7 (124.7)	310.0	900.0			699.2 (147.5) ^e^	470	1030
Birthweight (g)			3579.2 (424.4)	2297.0	4520.0			3549.5 (463.2)	2325	4760

*GA* gestational age in weeks

^a^4-vessel: based on self-reported pre-pregnancy weight and height, STORK: based on measured weight and height at first visit

^b^First trimester measurement

^c^4-vessel: no smoking during pregnancy vs. stopped smoking in first trimester. No women continued smoking once they were aware of their pregnancy, STORK: self-reported “no smoking during pregnancy” vs. “smoking”

^d^University college/university education (> 15 years)

^e^Missing data: 4 vessel cohort: pre-pregnancy BMI *n* = 72; STORK cohort: GA 1st sampling *n* = 64, placental weight *n* = 66

### Proteins released to and taken up from the maternal circulation by the placenta

Among the 4565 proteins, 256 proteins were released and 101 were taken up on the maternal side of the placenta. The results for all 4565 proteins are given in Additional file 2: Table S3.

Placental growth factor (PGF) showed a 76% higher level in the uterine vein as compared to the radial artery (Table 3) and was among the ten placenta-released proteins with the smallest FDR adjusted *p*-values. Also, on this list of placenta-derived proteins were pleiotrophin (PTN), the Wnt antagonist secreted frizzled-related proteins 1 and 3 (SFRP 1 and FRZB), and fatty-acid amide hydrolase 2 (FAAH2). Vascular endothelial growth factor receptor 1 (interpreted as sFlt-1), quantified by two SOMAmers, was also released from the placenta to the maternal circulation, with a 33–35% higher level in the uterine vein as compared to the radial artery (Additional file 2: Table S3).

**Table 3:** Placental release and uptake. The ten proteins with the smallest FDR adjusted *p*-values

Protein name	Entrez gene symbol	FDR adjusted *p*-value*	
Proteins released by the placenta to the maternal circulation			% higher RFU in uterine vein
Pleiotrophin	PTN	7.57E−17	33.68
Fatty-acid amide hydrolase 2	FAAH2	2.90E−16	97.85
Secreted frizzled-related protein 3	FRZB	2.90E−16	56.58
Secreted frizzled-related protein 1	SFRP1	2.90E−16	22.62
Signal peptide, CUB and EGF-like domain-containing protein 3	SCUBE3	4.08E−16	23.77
Placenta growth factor	PGF	1.17E−15	76.17
Urokinase-type plasminogen activator	PLAU	2.68E−14	55.37
Tubulin polymerization-promoting protein family member 2	TPPP2	2.49E−13	32.90
Noggin	NOG	2.49E−13	14.44
Transgelin	TAGLN	8.36E−13	38.39
Proteins taken up by the placenta from the maternal circulation			% lower RFU in uterine vein
Beta-defensin 1	DEFB1	3.14E−11	14.51
Insulin	INS	3.06E−07	6.53
Parathyroid hormone	PTH	5.95E−07	9.92
Urokinase plasminogen activator surface receptor	PLAUR	2.13E−06	6.19
Corticotropin-releasing factor-binding protein	CRHBP	7.53E−05	5.60
Trefoil factor 2	TFF2	8.12E−05	5.41
Vascular endothelial growth factor A, isoform 121	VEGFA	1.11E−04	4.19
Trefoil factor 1	TFF1	1.89E−04	6.09
Steroidogenic acute regulatory protein, mitochondrial	STAR	2.10E−04	6.25
Complexin-2	CPLX2	2.40E−04	3.05

^*^Paired *t*-tests of log 2 RFU venoarterial difference using Benjamini-Hochberg false discovery rate

Among the ten proteins taken up by the placenta on the maternal side with the smallest FDR adjusted *p*-values (Table 3), we found the antimicrobial agent β-defensin 1 (DEFB1) and parathyroid hormone (PTH) with a 14% and 10% lower level in the vein compared to the artery, respectively. Among proteins with the highest uptake from the maternal circulation to the placenta, phosphoglycerate mutase 1 (PGAM1) had 7% lower level in the uterine vein as compared to the radial artery.

The difference in protein levels between the vein and artery indicated higher relative (%) venoarterial difference for the proteins released as compared to taken up by the placenta.

### Placenta-specific released proteins to the maternal circulation

**Fig. 2:**
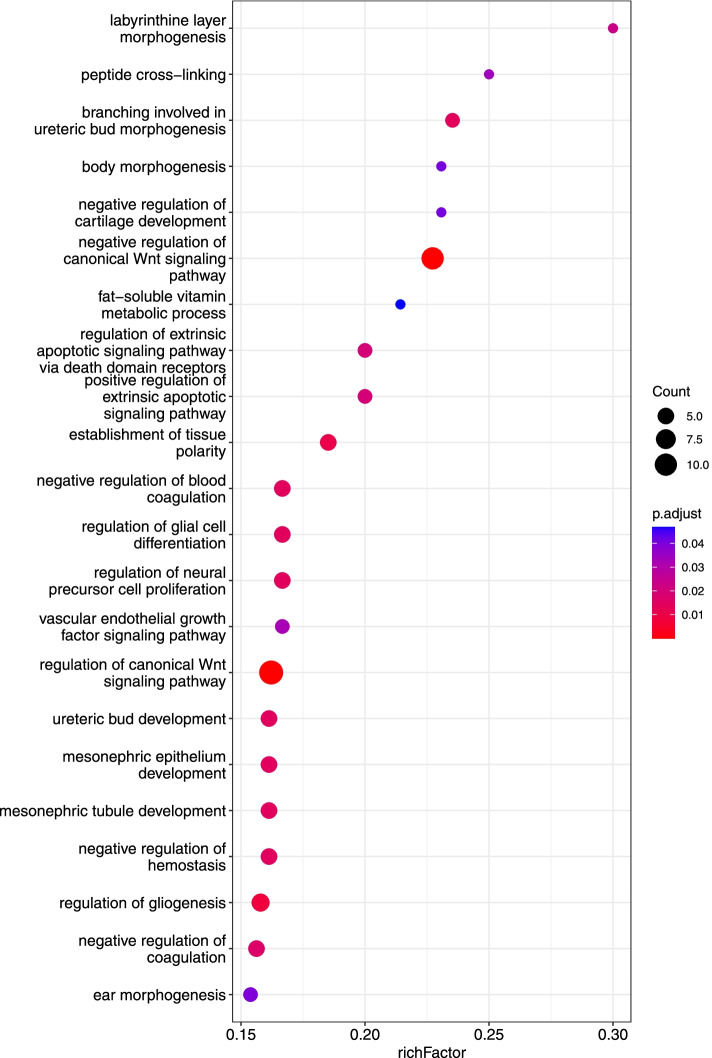
Gene ontology enrichment analysis of placenta-specific released proteins to the maternal circulation with a richFactor above 0.15

Two-thousand nine-hundred and forty-nine (2149) proteins were significantly different (both negative and positive) between the antecubital vein and the radial artery (arm vessels, proxy for the systemic venoarterial difference) (FDR < 0.05). To narrow in on placenta-specific released proteins, we compared the venoarterial differences between the uterine vein-radial artery (placenta) and the antecubital vein–radial artery (the arm) (Additional file 1: S1.3 and Table S2 for further explanations). We found 101 placenta-specific released proteins (Additional file 2: Table S3). Gene ontology enrichment analysis showed that enriched biological processes with a richFactor above 0.15 included labyrinthine layer morphogenesis, regulation of the WNT pathway, regulation of gliogenesis, regulation of coagulation and hemostasis, and the vascular endothelial growth factor signaling pathway (Fig. 2). The full enrichment result is available in Additional file 3: Table S4.

### Placenta-specific released proteins across gestation in the maternal circulation

**Fig. 3:**
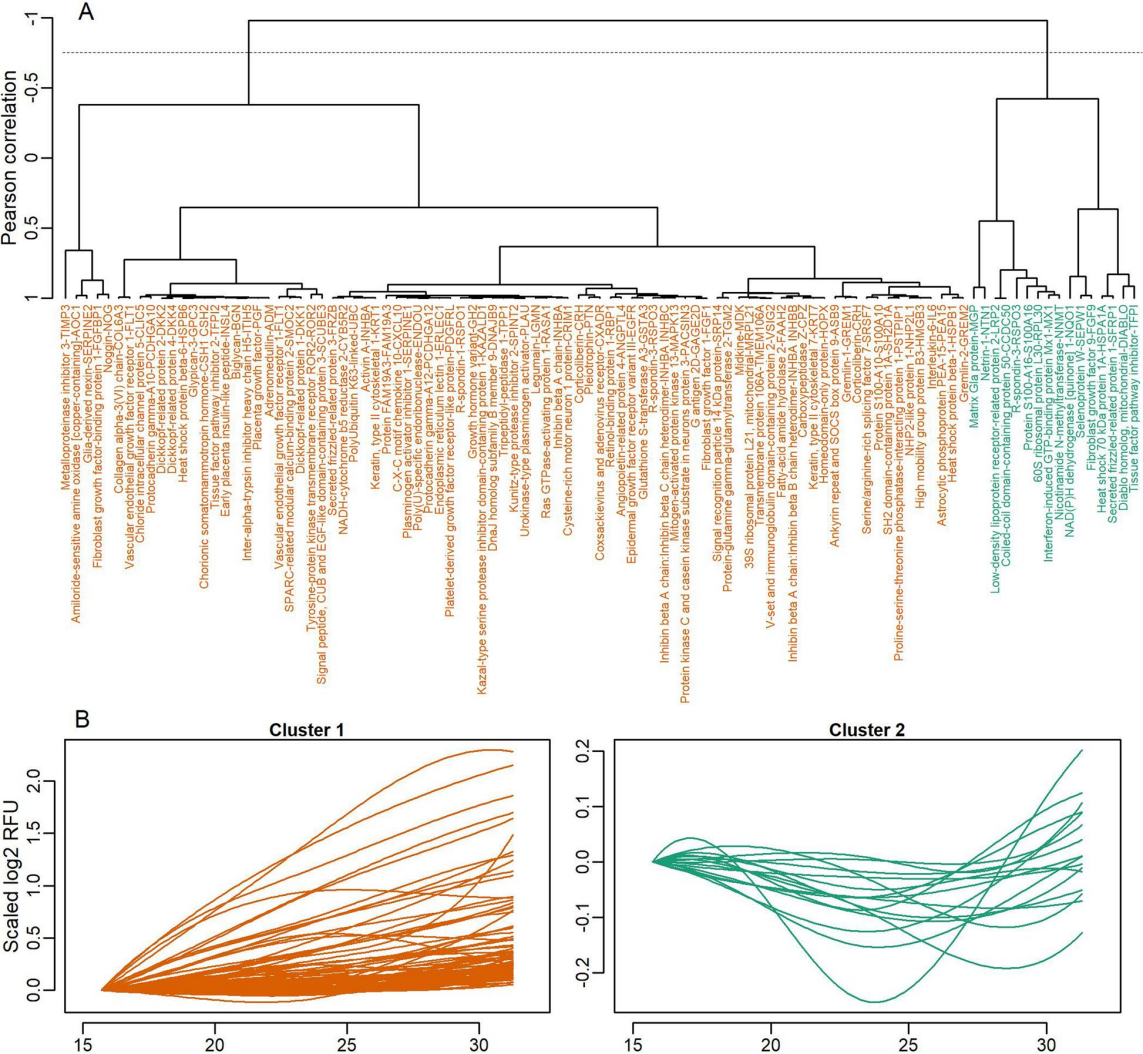
**A** Dendrogram and **B** longitudinal patterns for placenta-specific released proteins in the maternal circulation. **A** The horizontal dotted line indicates where the dendrogram was cut to create the two clusters. **B** Protein development over the three time points was scaled to start at zero within each cluster after cutting, and proteins from the same cluster were plotted in the same plots

The models of longitudinal changes of the 101 placenta-specific released proteins estimated 101 curves summarizing each protein’s mean development across gestation (Additional file 4: Fig. S2). The mean curves were clustered based on Pearson correlation. Silhouette analysis indicated that the optimal number of clusters was two (Fig. 3A). Figure 3B shows the changes in abundance in the maternal circulation across gestation of all proteins per cluster after scaling so that all proteins within each cluster have the starting log2 RFU at zero.

The largest cluster (cluster 1) consisted of 85 proteins and displayed a steady increase in levels across gestational age. Among proteins in this cluster were PGF, FLT1 (interpreted as sFlt-1), FRZB, PTN, TFPI2, and FAAH2. These results indicate that the majority of the placenta-specific proteins increase with gestational age. Proteins in cluster 2 showed a more heterogenous longitudinal pattern, with both decreasing and increasing trends across gestation.

### Placental proteomic clock

**Fig. 4:**
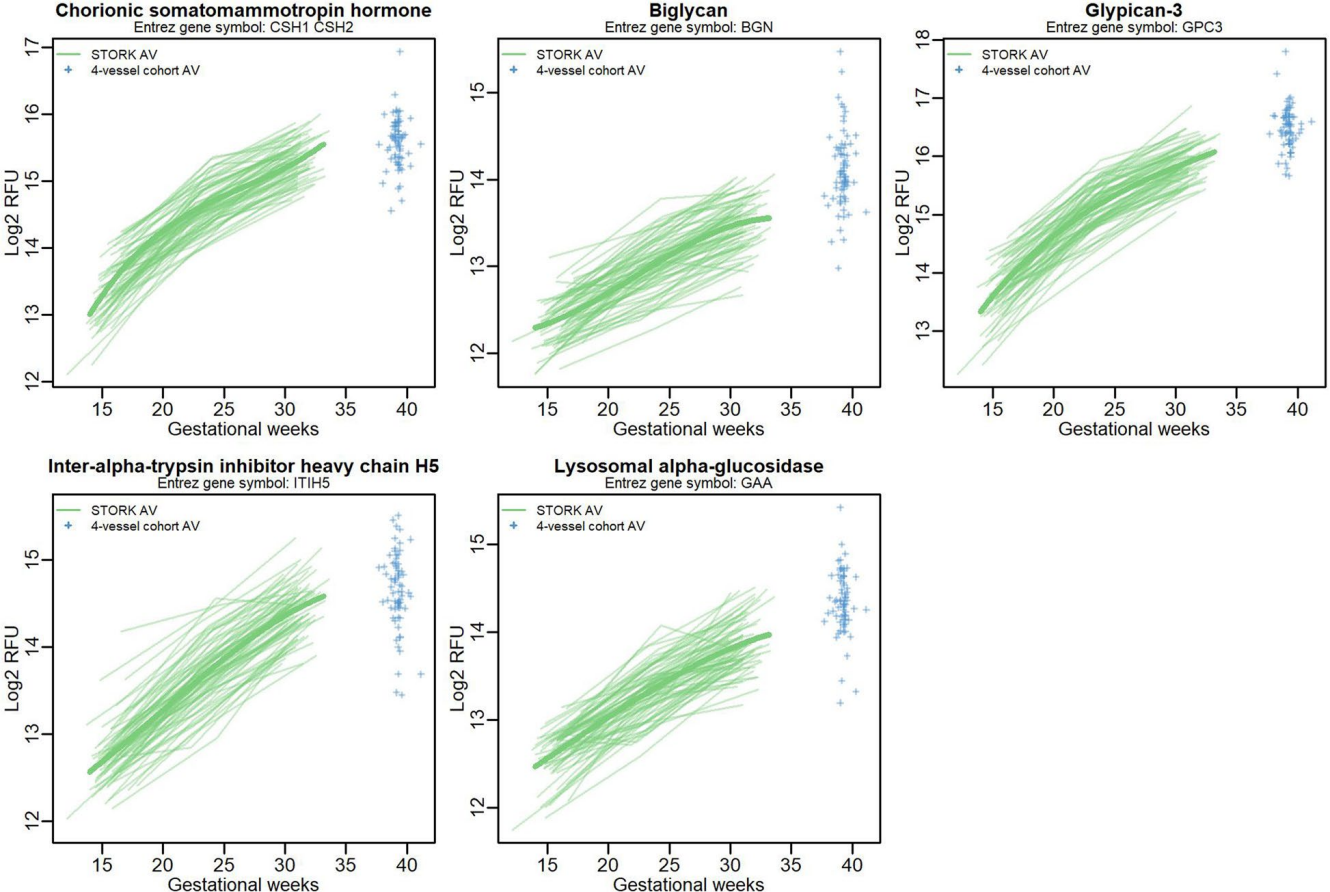
Line plots of placental proteomic clock proteins that tightly track gestational age based on STORK longitudinal samples; 4-vessel antecubital vein samples were added to the line plots only for visualization purposes and were not included in the analyses

**Fig. 5:**
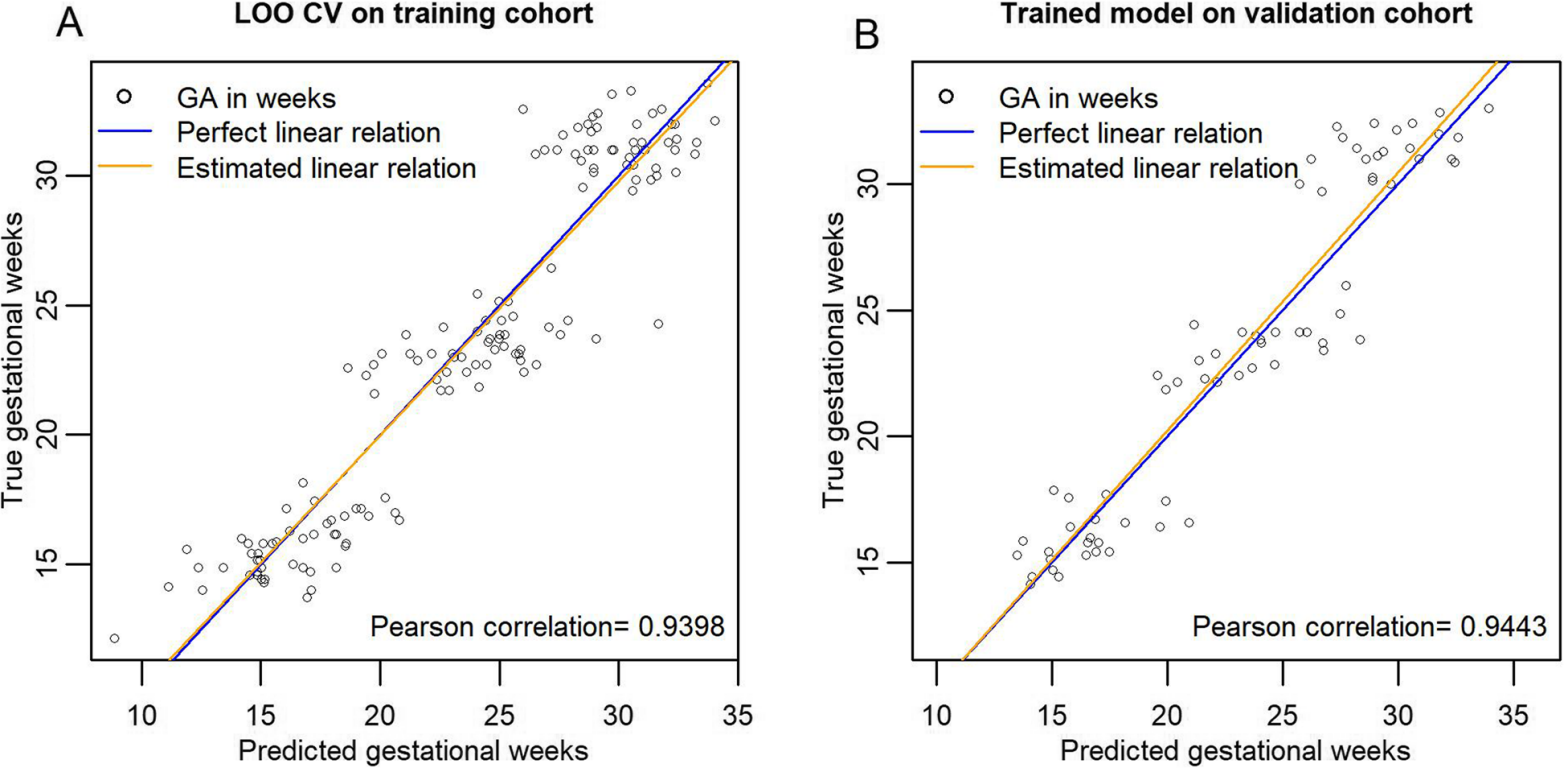
Performance of placental proteomic clock proteins in a linear model together with the clinical variables nulliparity, maternal BMI, and maternal age. Plot **A** shows performance based on leave-one-participant-out cross validation in the training cohort (2/3 of all STORK participant samples). Plot **B** shows performance of the trained linear model on the validation cohort (1/3 of STORK participant samples)

A machine learning algorithm, the variable selection algorithm elastic net with stability selection (ENSS), identified five placenta-derived proteins that could predict gestational age at the time of sampling in the same model. We termed the linear model including these proteins a “placental proteomic clock.” The placental proteomic clock consisted of chorionic somatomammotropin (CSH1/2, also known as placental lactogen), biglycan (BGN), glypican 3 (GPC3), inter-alpha-trypsin inhibitor heavy chain H5 (ITIH5), and lysosomal alpha-glucosidase (GAA) (Fig. 4). The Pearson correlation for the predicted versus the true gestional age at sampling was > 0.9 (Fig. 5). The final trained linear model equation was GA in weeks = −101.93028 + CSH1/2*0.40874 + BGN*2.04161 + GPC3*1.28050 + ITIH5*2.83024 + GAA*2.60163 + BMI*−0.9889 + Age*0.9807 + Nulliparity*−0.20379.

## Discussion

Using our 4-vessel sampling method combined with a 5000-multiplex proteomic platform, we found evidence of significant exchange of a large number of proteins between the maternal and placental compartments in vivo in healthy pregnancy near term. Furthermore, we narrowed in on proteins that exhibited enhanced release from the placenta (*placenta-specific released proteins*), by testing venoarterial difference in the placenta as compared to the systemic circulation. These proteins are interesting both in terms of placental and maternal physiology during pregnancy and as biomarkers of placental function since their abundance in the systemic circulation may be influenced by placental release. To the best of our knowledge, no other research groups have been able to perform such a detailed analysis of placenta-derived proteins in human pregnancy.

Due to our robust study design that combines the 4-vessel cohort and the STORK cohort, we were able to create longitudinal protein curves across gestation for the identified placenta-specific released proteins (Additional file 4: Fig. S2). These curves may serve as a first step towards a reference for the expected physiological longitudinal pattern of placenta-derived protein across gestation. However, there is large variation in abundance across gestation in the systemic circulation between the pregnant women. These inter-individual differences in protein abundance may indicate that establishment of absolute cut-off values for normal abundance of placenta-derived proteins can be difficult. Instead, we hypothesize that development of reference curves for protein abundance for a selected group of placental proteins may serve better as biomarkers of placental function. Such reference curves would allow each pregnant woman to be her own control using repeated measures of proteins, tracking protein development across gestation.

Using a machine learning algorithm, elastic net with stability selection, we identified five placenta-derived proteins (CSH, BGN, GPC3, ITIH5, and GAA) that followed a tight chronological profile across gestation when measured in the antecubital vein. The resulting *placental proteomic clock*, inspired by the work of Aghaeepour et al. [7], represents a novel concept where deviations from the expected gestational pattern of these placenta-derived proteins may serve as indication of placental dysfunction. The identified five placental proteomic clock proteins showed promising predictive ability when combined in a linear model, evident by prediction performance above 90% in both the training and the validation cohort. The abundance of these five placenta-derived proteins increased linearly with gestational age, possibly reflecting placental maturation or size. However, there was no correlation between placental weight and placental release (VA difference) for these five proteins (data not shown). Furthermore, even if the observed linear increase in abundance of these proteins is a reflection of placental maturation or size, the placental clock proteins may still be of value to track placental well-being and function. Interestingly, GPC3, BGN, and CSH1/2 were identified as clock proteins in both Aghaeepours and the current study [7]. Furthermore, Romero et al. also found that the proteoglycans GPC3 and BGN had a 26.04 and 2.62-fold increase across gestation, respectively [35]. Inter-alpha-trypsin inhibitor heavy chain H5 is involved in the dynamics of the extracellular matrix and has been linked to pregnancy and uterine development in animal studies [36]. Lysosomal alpha-glucosidase is an enzyme that breaks down glycogen in the lysosome with known expression in the placenta [14].

Several proteins identified in the current study as released from the placenta into the maternal circulation have ample evidence in the literature to suggest placental origin. Placental growth factor (PGF) was confirmed to be significantly released to the mother, which is consistent with our pilot study [16] and a former study from our group using ELISA for protein determination [3]. Furthermore, our longitudinal data show that the abundance of PGF in the maternal circulation increases over gestation until gestational week 30, similar to previous studies [37]. We believe that the placenta-derived protein identified by Entrez Gene Symbol FLT1 is the soluble fms-like tyrosine kinase-1 (sFlt1), based on aptamer binding to the extracellular amino acid sequence that is similar between FLT1 and sFlt1. Placental release of the soluble form of FLT1 to the maternal circulation in preeclamptic pregnancies has previously been shown by our group [3, 38] and in healthy pregnancies by others [39]. These observations validate the approach used in the current study.

Compared to our pilot study that showed 34 proteins significantly released into the maternal circulation using 4-vessel samples on a 1310 multiplex SomaScan platform [16], we now identified 256 proteins as released to the mother on the 5000 multiplex SomaScan platform. Among the 256 placenta-derived proteins released in the current study, 30 of the 34 identified placenta-derived proteins in the pilot study were confirmed (Additional file 1: Fig. S3). Thus, we identified 226 novel placenta-derived proteins. Several of the proteins listed as released by the placenta have not previously been described in humans to best of our knowledge (for example FAAH2, Heat shock 70 kDa protein 1A (HSPA1A) and Heat shock protein beta-6 (HSPB6)).

One-hundred and one proteins were taken up by the placenta according to the current study, whereas nine proteins were taken up in the pilot study [16]. Three of the nine proteins in the pilot study, including VEGFA (vascular endothelial growth factor A, isoform 121), trefoil factor 1 (TFF1), and urokinase plasminogen activator surface receptor (PLAUR), were confirmed in the current study. Two of the nine proteins identified as taken up in the pilot study were not included in the analysis based on the 5000-plex platform. Thus, the current study identified 92 novel proteins as taken up by the placenta as compared to the pilot study.

Differences in proteins identified as released and taken up in the current study as compared to our pilot study originates both from differences on the platform (more proteins included in 5000-plex platform) and more statistical power in the current study due to larger sample size, as well as data preprocessing steps. In the current study, we used “median normalization” [30] on the venoarterial differences to adjust for movement of water across the placenta and other potential biases influencing the data, whereas a fixed global factor was used to correct for any water shifts in the pilot study as described by Holm et al. [16, 19].

It is well known that the maternal plasma proteome changes across gestation [7, 35]. When interpreting our findings, it is important to take into consideration the cross-sectional nature of the 4-vessel sampling. Thus, the proteins we have defined as being released or taken up by the placenta are a reflection of physiology at the time of the cesarean section near term gestation. Our longitudinal analysis of the antecubital vein levels of placenta-specific released proteins across gestation showed two clusters with distinct patterns of change across gestation. We hypothesize that proteins with high abundance in early pregnancy have the most impact on physiology at an early stage, whereas the proteins that increase in abundance towards term play a larger role at the end of pregnancy. Some changes in abundance may reflect placental mass or maturation. Among the placenta-specific released proteins, the Wnt regulation was prominent (Fig. 2). This finding is in accordance with data showing that Wnt signaling plays important roles in normal physiology and abnormal trophoblast function [40]. Interestingly, GO biological function labyrinthine layer morphogenesis was enriched. Rinkenberger and Werb stated “the labyrinth in the mouse and the floating chorionic villi in human are homologous structures, both characterized by extensive branching” [41]. Thus, this GO term is also used in humans, and our finding may indicate active morphogenesis in the human placenta close to term.

**Fig. 6:**
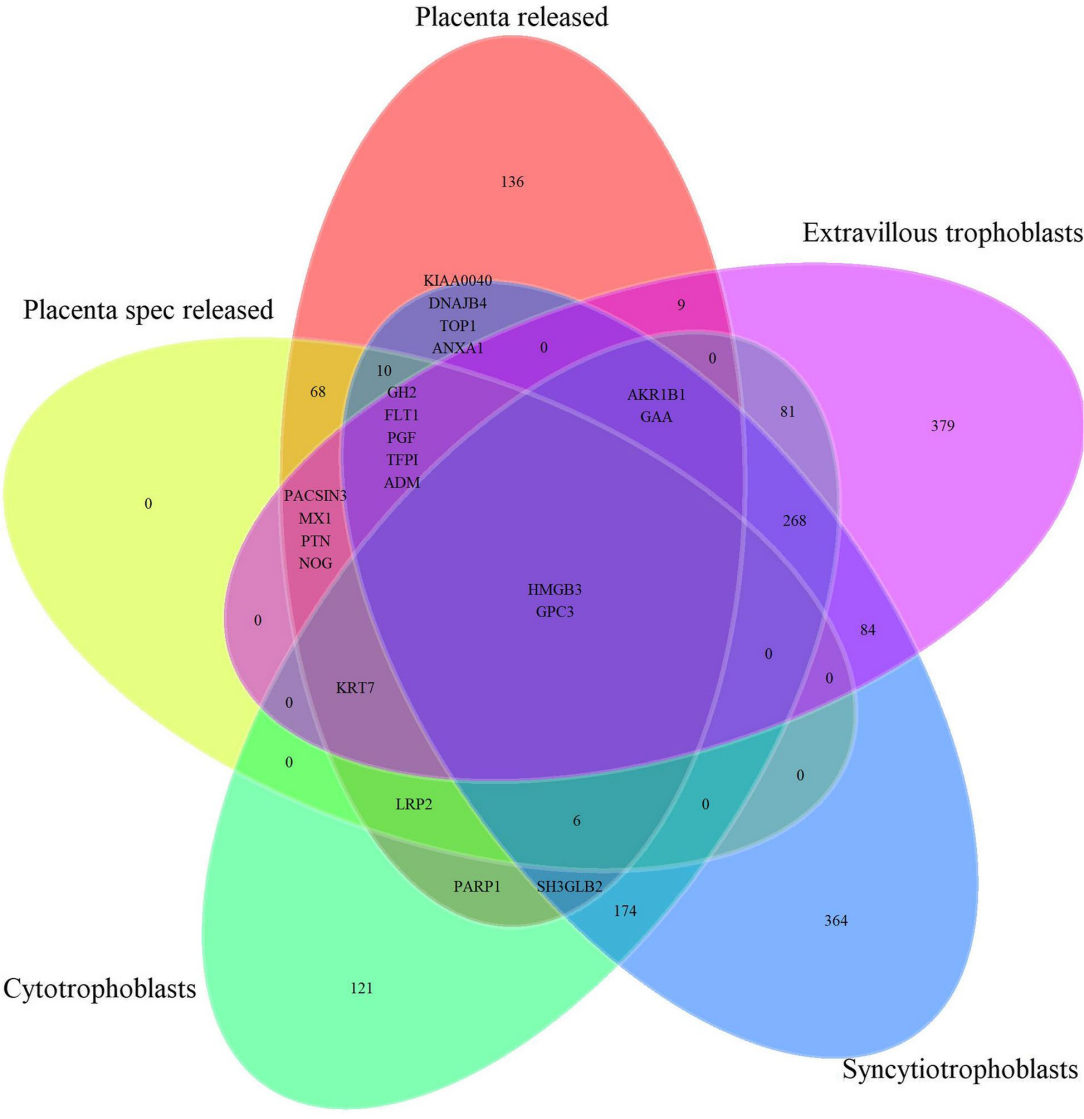
Overlap between proteins defined as released by the placenta and placenta-specific released in the current study mapped against proteins identified as elevated in three types of trophoblast cells in The Human Protein Atlas data. Placenta spec release = placenta-specific release

This study has some strengths and limitations. We have used the term “placenta” being well aware that the uterine vein, from which samples were taken, also drains non-placental tissues like decidua, myometrium, and chorion. Furthermore, we use the radial artery as a proxy for the uterine artery. The reason we use “placenta” is partly that the conceptual aim is placental functions, partly for the sake of simplicity. We acknowledge that proteins may enter the circulation in different ways, including exocytosis, or by random shedding or apoptosis of cells. Thus, some proteins found to be released by the placenta could be due to non-secretory processes. Within the placenta, there are a variety of cells that may contribute to the proteins released, including syncytiotrophoblasts, cytotrophoblasts, mesenchymal stem cells, fibroblasts, and immune cells. Twenty-three percent (3008 /13074) of proteins expressed in the placenta according to the Human Protein Atlas are measured by the SomaLogic 5000 multiplex platform (Additional file 1: Fig. S4A). Furthermore, 37.5% (108/288) proteins that have been shown to have elevated expression in the placenta as compared to other tissues in the Protein Atlas database are measured by the 5000-mulitplex platform. Additional file 1: Fig. S4B shows that most of the placenta-derived and placenta-specific proteins overlap with the 13074 proteins expressed in the placenta from Human Protein Atlas. Additionally, 19 of our 256 placenta-derived proteins overlap with the 108 elevated expressed proteins according to the Human Protein Atlas (data not shown). Mapping of placenta-derived proteins and placenta-specific released proteins in our study to genes with elevated expression in syncytiotrophoblasts, cytotrophoblasts, and extravillous trophoblasts according to the Proteins Atlas show considerable overlap (Fig. 6).

Traditional proteomics has shortcomings in terms of the need to remove highly abundant proteins as well as limited capacity to identify a broad range of proteins. The SOMAscan protein-binding technology offers the possibility to investigate protein expression on a platform with high sensitivity and dynamic range for a large number of specific proteins, although the quantification is in relative fluorescence levels. Absolute quantification of the proteins could have provided additional information, but the use of the relative protein levels is sufficient when comparing vessels. However, absolute quantification may improve the clinical relevance of longitudinal reference curves for placenta-derived proteins. The proteins have been selected to include human proteins associated with disease, and the aptamer technology therefore lacks the unbiased discovery approach character.

We performed a median normalization [30, 31] of venoarterial differences in the placenta (uterine vein-radial artery) based upon the assumption that most proteins are not different between the vein and artery. This assumption takes advantage of the power of the nearly 5000 proteins measured and adjusts for any movement of water across the placenta and other possible biases that may affect venoarterial differences (see methods). However, there is a risk that this method resulted in too many venoarterial protein differences set to zero, excluding biologically relevant proteins from our findings.

The SomaScan was performed on the antecubital vein samples in the 4-vessel cohort with the intention of using the venoarterial differences in the arm (antecubital vein vs radial artery) as a negative control of venoarterial differences in “the placenta” (radial artery vs uterine vein). We used this negative control to define proteins especially released from the placenta as “placenta-specific proteins.” Interestingly, we found that a large number of proteins had different levels in the antecubital vein and radial artery, possibly reflecting protein exchange in musculoskeletal tissues or systemic differences between veins and arteries. We are well aware that strictly all venoarterial differences in any maternal tissues or organs should have been tested as negative controls before concluding that proteins released by placenta are specific [3]. However, our method is, to the best of our knowledge, the only feasible one. Additionally, our definition of proteins released by the placenta as “placenta-specific” combined with a false discovery rate of 5% mean that our analysis followed strict criteria.

## Conclusions

Using a human in vivo study, we identified 256 proteins released and 101 proteins taken up by the placenta that may guide future studies investigating placental physiology and pathology. The identified placenta-derived proteins can be measured in the systemic maternal circulation and may serve as biomarker candidates of placental function. Among the placenta-derived proteins, we identified five “*placental proteomic clock*” proteins that were tightly linked to gestational age. We introduce the novel concept where deviating developmental patterns of placenta-derived proteins across gestation may be used to indicate placental dysfunction. This “placental proteomic clock” concept warrants further investigation.

## Supplementary Information

**Additional file 1: Table S1.** The number of plasma samples included in the analyses from both cohorts. **Figure S1.** P-value histogram from t-tests between uterine vein and radial artery (maternal side of the placenta) showing an overabundance of very low *p*-values along with a uniform distribution of higher *p*-values, indicating that statistical testing assumptions are met, experimental bias is minimal and that we can expect true positive results. **Table S2.** Criteria 1 or 2 define placenta-specific release of proteins to the maternal circulation. **Figure S3.** Proteins released to the maternal circulation according to the pilot study (1310 proteins measured) and the current study (4979 proteins measured). In the current study, 226 novel placenta-derived proteins were identified. **Figure S4.** A. Overlap between proteins measured on the current SomaScan (5000 plex) and the former SomaScan (1310 plex) with Human Protein Atlas data on proteins that are either expressed in the placenta or have elevated expression in the placenta. B. Overlap between results from current study; the 256 proteins released from the placenta and the 101 placenta-specific released proteins, and proteins expressed in the placenta tissue in general based on Human Protein Atlas data.

**Additional file 2: Table S3.** Complete overview of test results for all proteins, including placenta-derived proteins and placenta-specific released proteins.

**Additional file 3: Table S4.** Table of all enrichment results using placenta-specific released proteins.

**Additional file 4: Figure S2.** Longitudinal patterns of placenta-specific released proteins.

## Data Availability

The datasets used and/or analyzed during the current study are available from the corresponding author on reasonable request.

## Abbreviations

AV: Maternal antecubital vein
FDR: False discovery rate
GO: Gene ontology
PGF: Placental growth factor
RA: Radial artery
RFUs: Relative fluorescence units
sFlt-1: Fms-like tyrosine kinase 1
UV: Uterine vein
VEGFR: Vascular endothelial growth factor receptor
Wnt: Wingless-related integration site

## Footnotes

1 Script can be found at https://github.com/trondmic/Omics_in_pregnancy_OUS/blob/main/Proteomics_healthy_placenta/Proteins_released_and_taken_up.R

2 Script can be found at https://github.com/trondmic/Omics_in_pregnancy_OUS/blob/main/Proteomics_healthy_placenta/Median_normalization.R

3 Script can be found at https://github.com/trondmic/Omics_in_pregnancy_OUS/blob/main/Proteomics_healthy_placenta/Placenta_specific_across_gestation.R

4 Script can be found at https://github.com/trondmic/Omics_in_pregnancy_OUS/blob/main/Proteomics_healthy_placenta/GO_of_specific_proteins.R

5 Script can be found at https://github.com/trondmic/Omics_in_pregnancy_OUS/blob/main/Proteomics_healthy_placenta/Placental_proteomic_clock.R
