# Supplementary material for: Placenta-derived proteins across gestation in healthy pregnancies—a novel approach to assess placental function?
Source: BMC Med. 2022 Jul 1;20:227. doi: 10.1186/s12916-022-02415-z (PMC9248112; doi:10.1186/s12916-022-02415-z)
Supplement: Supplementary file 1 — Additional file 1: Table S1. The number of plasma samples included in the analyses from both cohorts. Figure S1. P-value histogram from t-tests between uterine vein and radial artery (maternal side of the placenta) showing an overabundance of very low p-values along with a uniform distribution of higher p-values, indicating that statistical testing assumptions are met, experimental bias is minimal and that we can expect true positive results. Table S2. Criteria 1 or 2 define placenta-specific release of proteins to the maternal circulation. Figure S3. Proteins released to the maternal circulation according to the pilot study (1310 proteins measured) and the current study (4979 proteins measured). In the current study, 226 novel placenta-derived proteins were identified. Figure S4. A. Overlap between proteins measured on the current SomaScan (5000 plex) and the former SomaScan (1310 plex) with Human Protein Atlas data on proteins that are either expressed in the placenta or have elevated expression in the placenta. B. Overlap between results from current study; the 256 proteins released from the placenta and the 101 placenta-specific released proteins, and proteins expressed in the placenta tissue in general based on Human Protein Atlas data. [file 12916_2022_2415_MOESM1_ESM.docx]

***Placenta-derived proteins across gestation in healthy pregnancies – a novel approach to assess placental function?***

# **Additional file 1**

**Table S1.** The number of plasma samples included in the analyses from both cohorts.

|  | **Antecubital vein** | **Radial artery** | **Uterine vein** | **Antecubital vein**  **Time point 1** | **Antecubital vein**  **Time point 2** | **Antecubital vein**  **Time point 3** |
| --- | --- | --- | --- | --- | --- | --- |
| **4-vessel cohort** | 70 | 74 | 74 |  |  |  |
| **STORK cohort** |  |  |  | 70 | 70 | 70 |

**S1. Extended methods
S1.1 Protein quantification by SomaLogic**

To quantify proteins, we used SomaLogic’s microarray-based proteomic quantification platform called SomaScan assay version 4.0. There is one microarray per sample, and the microarrays consist of 4979 different types of probes that hybridize specifically to the corresponding aptamer. SomaLogic’s specialized type of aptamers are called Slow Off-rate Modified Aptamer reagents (SOMAmers). The 4979 unique SOMAmers bind specifically to one location on one specific protein. SOMAscan quantifies the fluorescence signal intensity of probes that have hybridized with SOMAmers from SOMAmer-protein complexes with specific binding. SomaLogic perform data standardization and quality control processing by use of control samples and control aptamers (Hybridization Control SOMAmer Reagents) that they add and analyze in parallel with the biological samples.

To ensure a large dynamic range, proteins are quantified in three different dilutions (diluted 1:5, 1:200 and 1:20000) of each biological sample. The dynamic range of the SomaScan assay has also improved from 10^8^ in the 1310-plex platform to 10^10^ in the 5000-plex platform [1]. Median intra- and interassay coefficients of variation are ~5% [1, 2] . Specificity of the SOMAmers has been established in several ways. Among 73 % of the aptamers, there was no detected binding to any related proteins of the target protein (1,612 aptamers) [3]. When binding to related proteins was detected, about half of these aptamers exhibited binding to at least one related protein with similar affinity while the other half bound to related proteins, but with at least tenfold weaker affinity [3].

Each SOMAmer has a unique structure and bind to one location per protein specifically [1]. However, two SOMAmers may bind to their specific locations on the same protein. The protein code (Entrez or Uniprot) for these SOMAmers will be identical. Furthermore, due to the high specificity of the Somalogic platform, we were able to identify different variants (for example isoforms) of different proteins. As an example, Inhibin beta A chain and Activin A, have separate unique aptamers, but identical Uniprot (P08476) and Entrez gene name (INHBA). This creates duplicated protein codes in our data set, as well as duplicated measurements and statistical tests.

**S1.2 P-value histograms**

Shapes of p-value histograms were used to diagnose experimental bias and the amount of false positives. We performed 4564 t-tests, which each require common statistical assumptions about our data. The results from our t-tests may be biased if these assumptions are not met. A uniform distribution of the histogram (equal amount of all p-values from 0 to 1) indicated that all individual null hypotheses were true (global null hypothesis is true) [4]. This means that assumptions were met, there was no dominating experimental bias and the venoarterial difference was truly 0 for all proteins, which means that p-values below any chosen cutoff (for example p-value of 0.05) were only false positives. However, histograms showing an overabundance of very low p-values and still a uniform distribution of higher p-values indicated the same as for uniformly distributed histograms, except that not all null hypotheses were true (the global null hypothesis may be rejected) and we expected true positives. See Breheny *et.al.* [4] for more details on p-value histograms. The histograms are given in Figure S1.


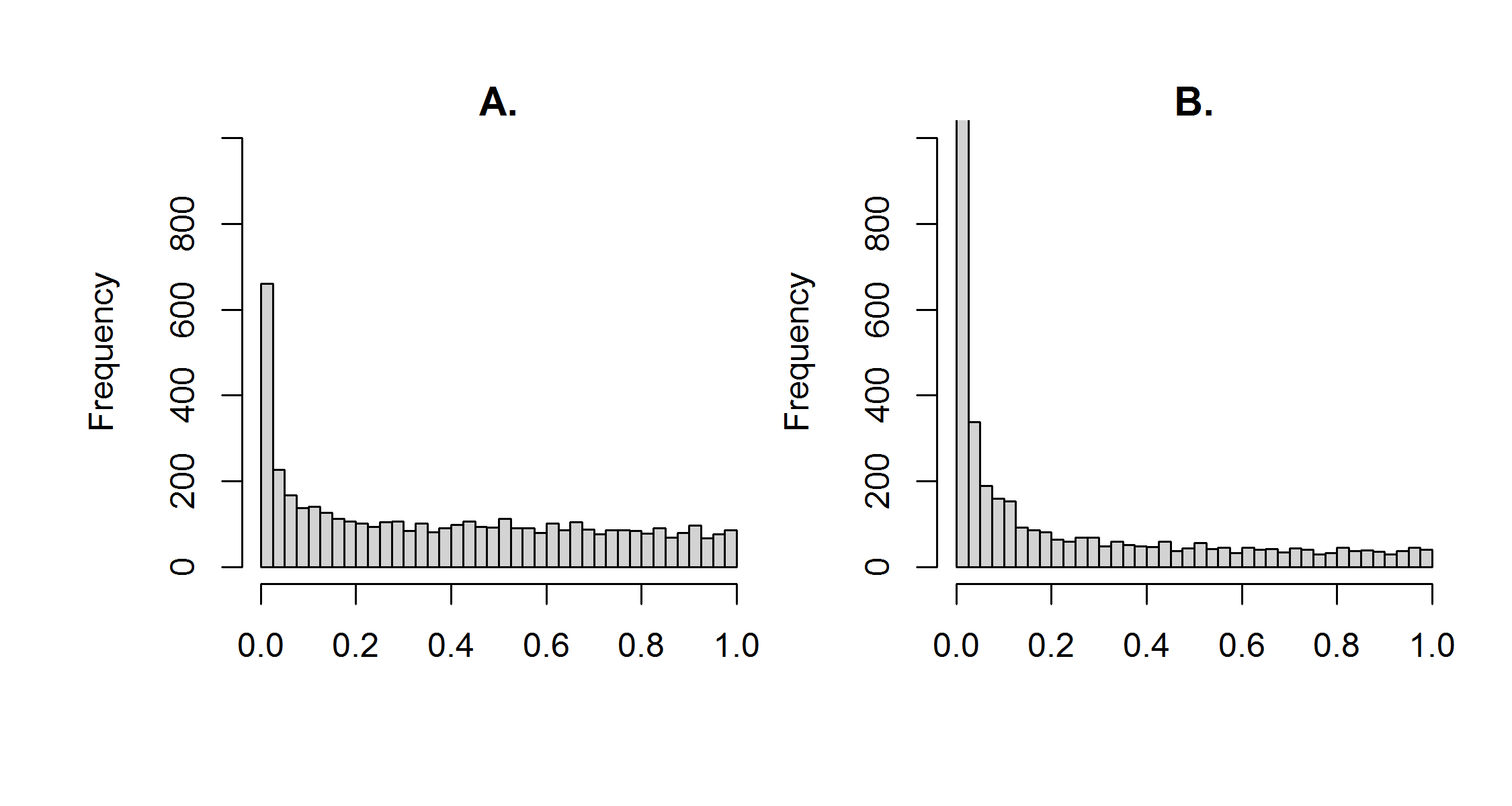


**Figure S1**. *P-value histogram from t-tests between uterine vein and radial artery (maternal side of the placenta) showing an overabundance of very low p-values along with a uniform distribution of higher p-values, indicating that statistical testing assumptions are met, experimental bias is minimal and that we can expect true positive results.*

**S1.3 Placenta-specific released proteins**

To narrow in on proteins with special relevance as placental proteins, we performed another set of t-tests comparing the venoarterial log2 RFU difference in the placenta (uterine vein-radial artery) with the arm (antecubital vein – radial artery) (Table S2).

**Table S2.** *Criteria 1 or 2 define placenta-specific release of proteins to the maternal circulation.*

| Placenta- specific release,  criteria | **Type of statistical tests per protein** | **Criteria per test** |
| --- | --- | --- |
| 1 | Two-sided Welch’s t-test for H_0_:  $log2\left( {RFU}_{\mathrm{UV}} \right)-log2\left( {RFU}_{\mathrm{RA}} \right)=0$  and  Two-sided Welch’s t-test for H_0_:  $(log2\left( {RFU}_{UV} \right)-log2\left( {RFU}_{RA} \right))$  $-$  $(log2\left( {RFU}_{\mathrm{AV}} \right)-log2\left( {RFU}_{\mathrm{RA}} \right))$  = 0 | - FDR-adjusted p-value < 0.05 - t statistic > 0   and   - FDR-adjusted p-value < 0.05 - t statistic > 0 |
| 2 | Two-sided Welch’s t-test for H_0_:  $log2\left( {RFU}_{\mathrm{UV}} \right)-log2\left( {RFU}_{RA} \right)=0$  and  Two-sided Welch’s t-test for H_0_:  $log2\left( {RFU}_{\mathrm{AV}} \right)-log2\left( {RFU}_{RA} \right)$= 0  or  $log2\left( {RFU}_{\mathrm{AV}} \right)<log2\left( {RFU}_{\mathrm{RA}} \right)$ | - FDR-adjusted p-value < 0.05 - t statistic > 0   and   - p-value > 0.05   or   - t statistic < 0 |

UV = uterine vein, RA = radial artery, AV =Antecubital vein

*
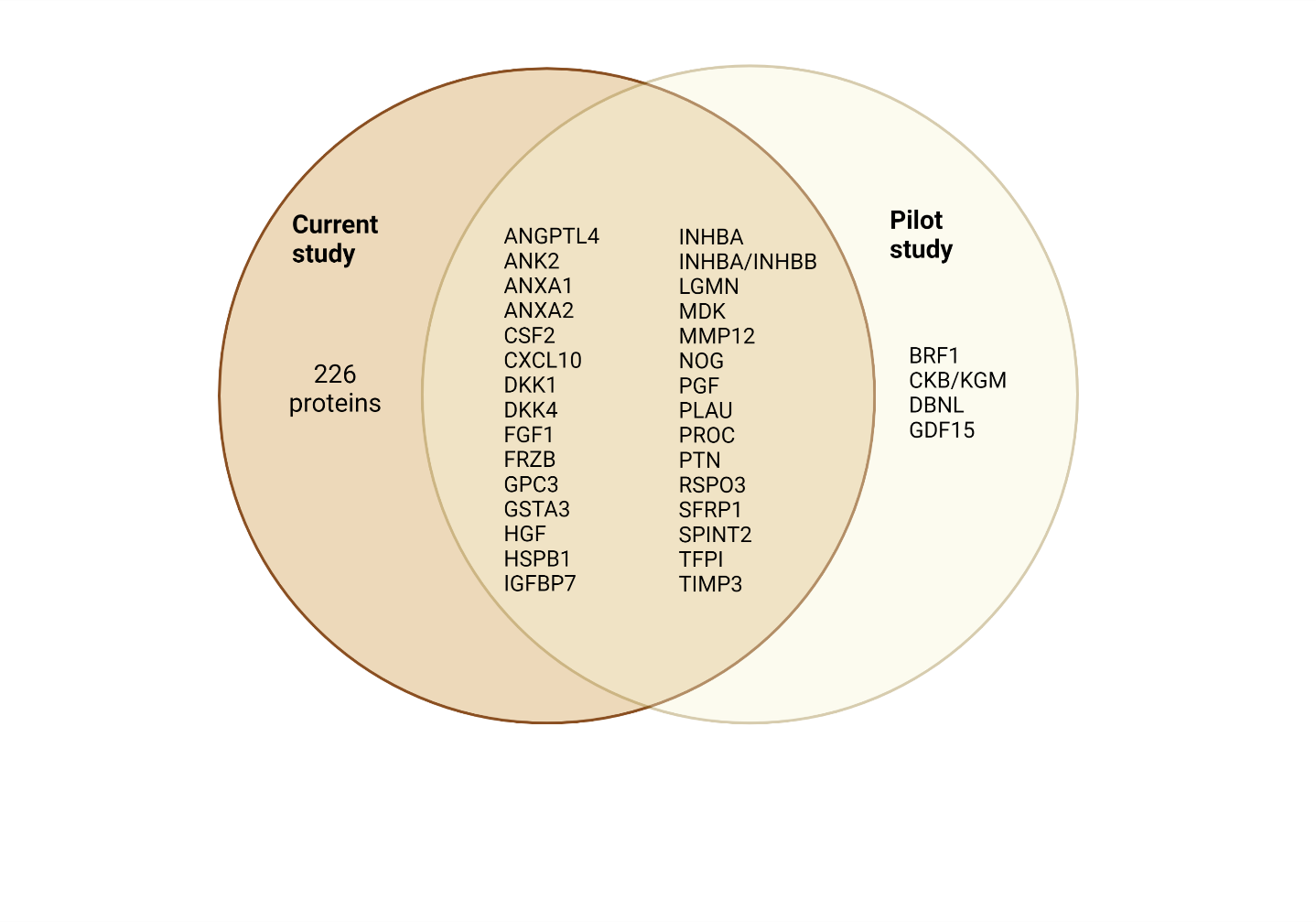
*

*
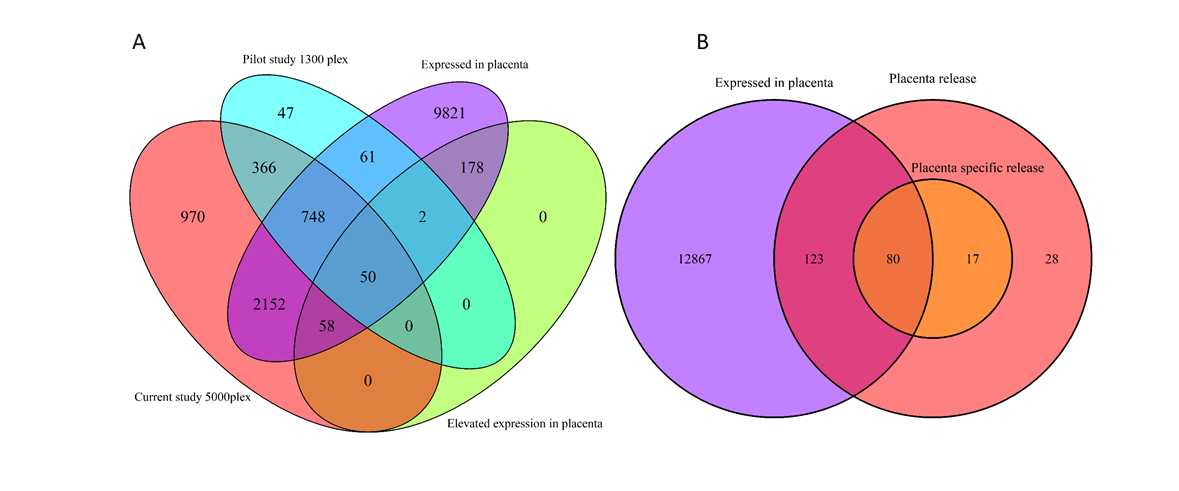
****Figure S3.*** *Proteins released to the maternal circulation according to the pilot study (1310 proteins measured) and the current study (4979 proteins measured). In the current study, 226 novel placenta-derived proteins were identified.*

***Figure S4****.* ***A:*** *Overlap between proteins measured on the current SomaScan (5000 plex) and the former SomaScan (1300 plex) with Human Protein Atlas data on proteins that are either expressed in the placenta or have elevated expression in the placenta.* ***B:*** *Overlap between results from current study; the 256 proteins released from the placenta and the 101 placenta-specific released proteins, and proteins expressed in the placenta tissue in general based on Human Protein Atlas data.*

References

1. SomaLogic Inc. SomaScan Assay v4.0 Technical Note. 2021.

2. Candia J, Cheung F, Kotliarov Y, Fantoni G, Sellers B, Griesman T et al. Assessment of Variability in the SOMAscan Assay. Sci Rep. 2017; 7(1):14248.

3. Williams SA, Kivimaki M, Langenberg C, Hingorani AD, Casas JP, Bouchard C et al. Plasma protein patterns as comprehensive indicators of health. Nat Med. 2019; 25(12):1851-1857.

4. Breheny P, Stromberg A, Lambert J. p-Value Histograms: Inference and Diagnostics. High Throughput. 2018; 7(3).
