# Supplementary material for: Placenta-derived proteins across gestation in healthy pregnancies—a novel approach to assess placental function?
Source: BMC Med. 2022 Jul 1;20:227. doi: 10.1186/s12916-022-02415-z (PMC9248112; doi:10.1186/s12916-022-02415-z)
Supplement: Supplementary file 4 — Additional file 4: Figure S2. Longitudinal patterns of placenta-specific released proteins. [file 12916_2022_2415_MOESM4_ESM.pdf]

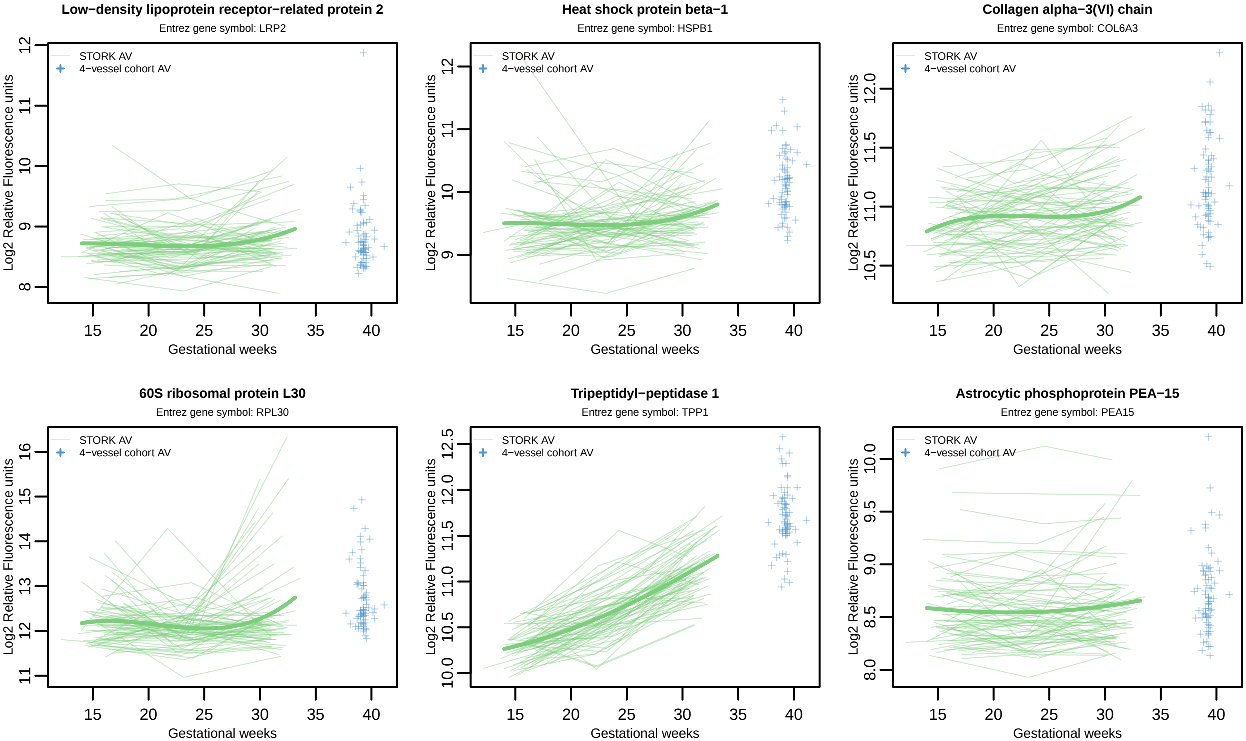
Low−density lipoprotein receptor−related protein 2
Entrez gene symbol: LRP2
Heat shock protein beta−1
Entrez gene symbol: HSPB1
Collagen alpha−3(VI) chain
Entrez gene symbol: COL6A3
60S ribosomal protein L30
Entrez gene symbol: RPL30
Tripeptidyl−peptidase 1
Entrez gene symbol: TPP1
Astrocytic phosphoprotein PEA−15
Entrez gene symbol: PEA15
STORK AV
4−vessel cohort AV
Log2 Relative Fluorescence units
Gestational weeks
15
20
25
30
35
40

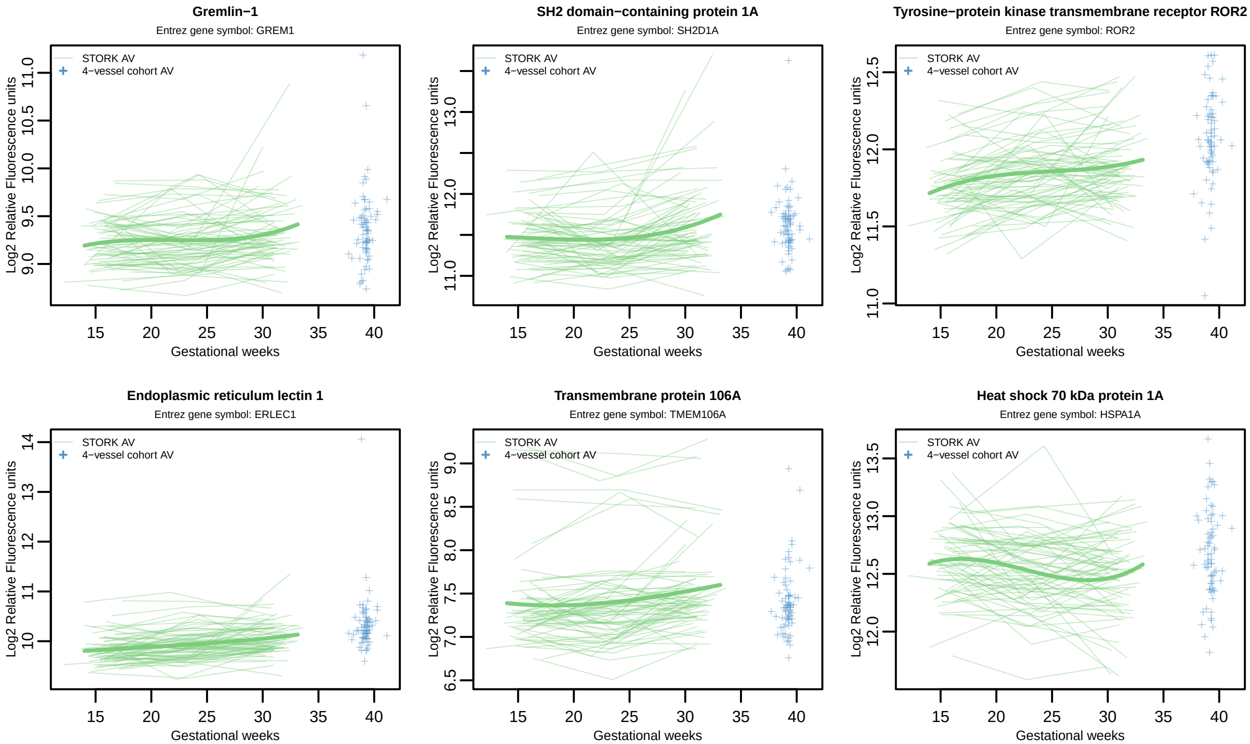
Gremlin−1
Entrez gene symbol: GREM1
SH2 domain−containing protein 1A
Entrez gene symbol: SH2D1A
Tyrosine−protein kinase transmembrane receptor ROR2
Entrez gene symbol: ROR2
Endoplasmic reticulum lectin 1
Entrez gene symbol: ERLEC1
Transmembrane protein 106A
Entrez gene symbol: TMEM106A
Heat shock 70 kDa protein 1A
Entrez gene symbol: HSPA1A
STORK AV
4−vessel cohort AV
Log2 Relative Fluorescence units
Gestational weeks
15
20
25
30
35
40

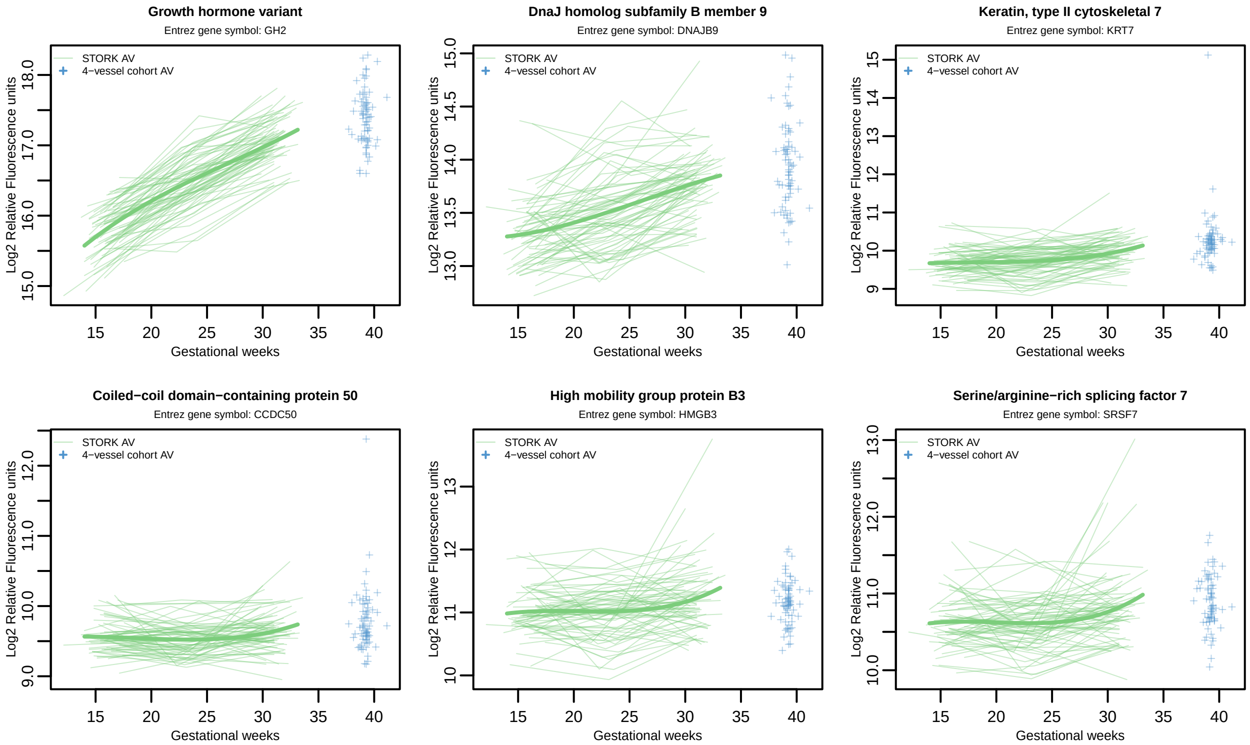
Growth hormone variant
Entrez gene symbol: GH2
DnaJ homolog subfamily B member 9
Entrez gene symbol: DNAJB9
Keratin, type II cytoskeletal 7
Entrez gene symbol: KRT7
Coiled−coil domain−containing protein 50
Entrez gene symbol: CCDC50
High mobility group protein B3
Entrez gene symbol: HMGB3
Serine/arginine−rich splicing factor 7
Entrez gene symbol: SRSF7
STORK AV
4−vessel cohort AV
Log2 Relative Fluorescence units
Gestational weeks
15
20
25
30
35
40

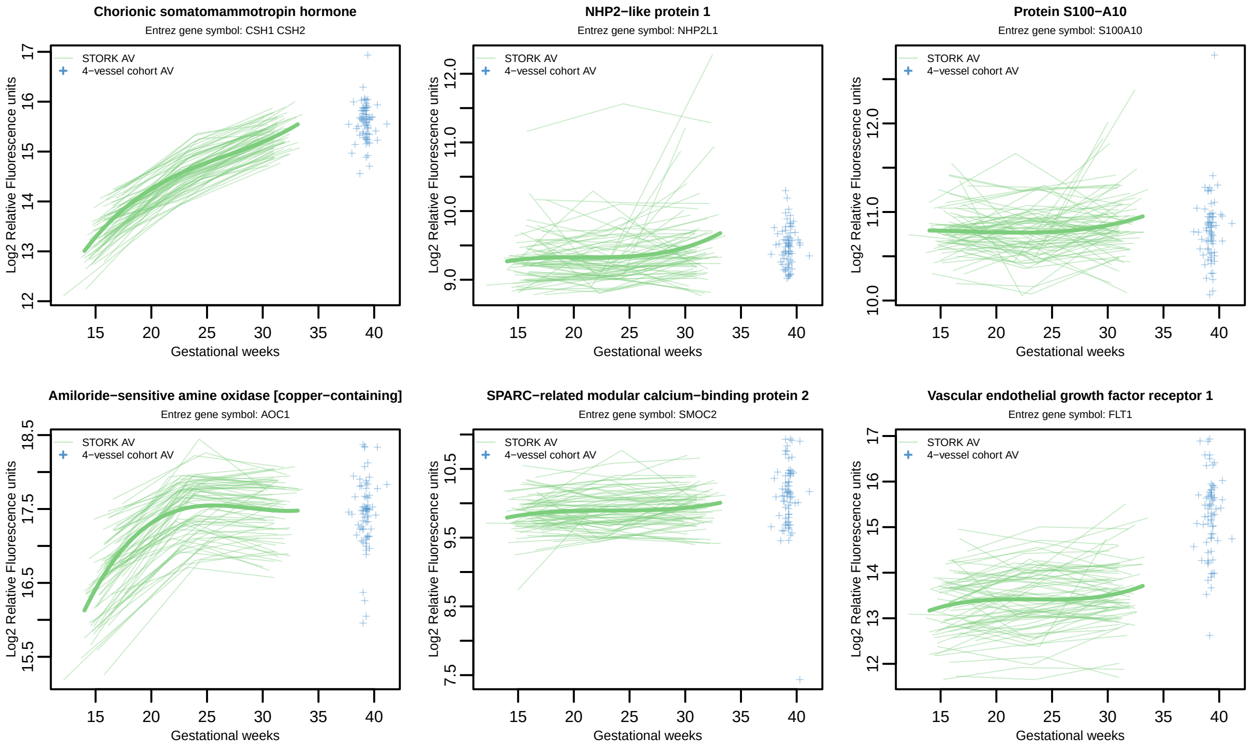
Chorionic somatomammotropin hormone
Entrez gene symbol: CSH1 CSH2
STORK AV
4−vessel cohort AV
Log2 Relative Fluorescence units
12
13
14
15
16
17
15
20
25
30
35
40
Gestational weeks
NHP2−like protein 1
Entrez gene symbol: NHP2L1
STORK AV
4−vessel cohort AV
Log2 Relative Fluorescence units
9.0
10.0
11.0
12.0
15
20
25
30
35
40
Gestational weeks
Protein S100−A10
Entrez gene symbol: S100A10
STORK AV
4−vessel cohort AV
Log2 Relative Fluorescence units
10.0
11.0
12.0
15
20
25
30
35
40
Gestational weeks
Amiloride−sensitive amine oxidase [copper−containing]
Entrez gene symbol: AOC1
STORK AV
4−vessel cohort AV
Log2 Relative Fluorescence units
15.5
16.5
17.5
18.5
15
20
25
30
35
40
Gestational weeks
SPARC−related modular calcium−binding protein 2
Entrez gene symbol: SMOC2
STORK AV
4−vessel cohort AV
Log2 Relative Fluorescence units
7.5
8.5
9.5
10.5
15
20
25
30
35
40
Gestational weeks
Vascular endothelial growth factor receptor 1
Entrez gene symbol: FLT1
STORK AV
4−vessel cohort AV
Log2 Relative Fluorescence units
12
13
14
15
16
17
15
20
25
30
35
40
Gestational weeks

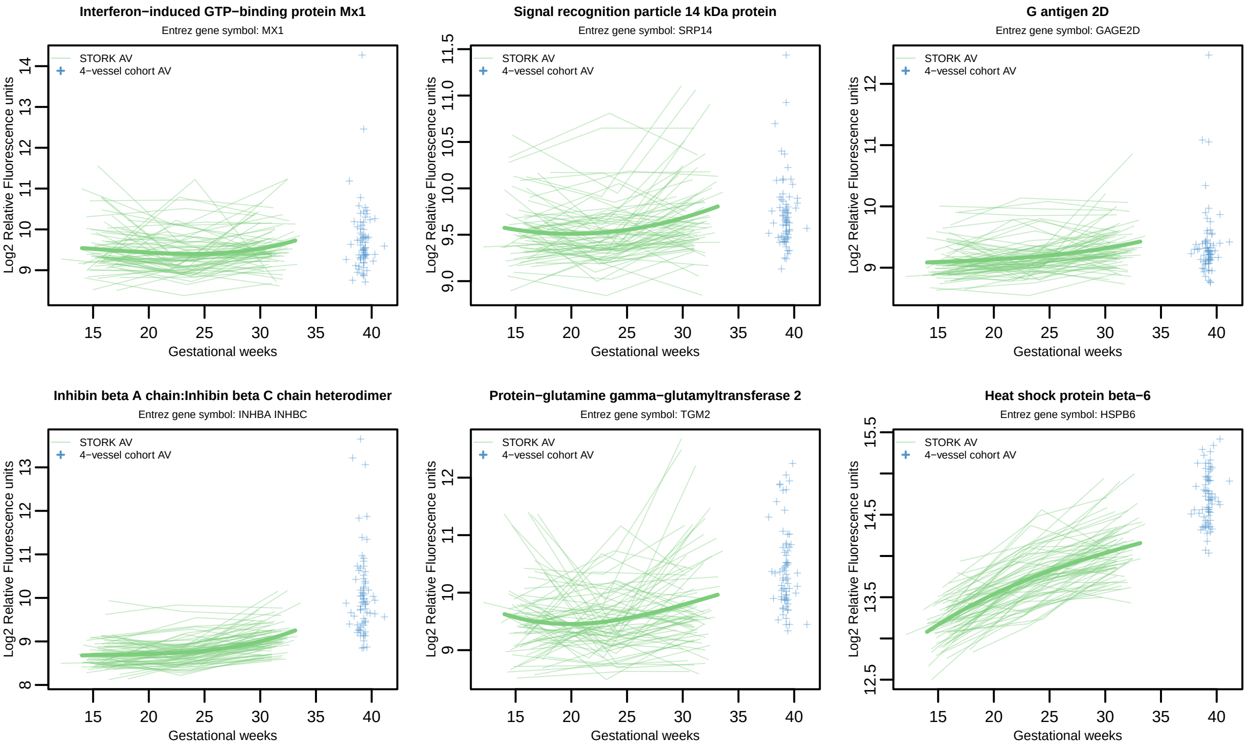
Interferon−induced GTP−binding protein Mx1
Entrez gene symbol: MX1
Signal recognition particle 14 kDa protein
Entrez gene symbol: SRP14
G antigen 2D
Entrez gene symbol: GAGE2D
Inhibin beta A chain:Inhibin beta C chain heterodimer
Entrez gene symbol: INHBA INHBC
Protein−glutamine gamma−glutamyltransferase 2
Entrez gene symbol: TGM2
Heat shock protein beta−6
Entrez gene symbol: HSPB6
STORK AV
4−vessel cohort AV
Log2 Relative Fluorescence units
Gestational weeks
15
20
25
30
35
40

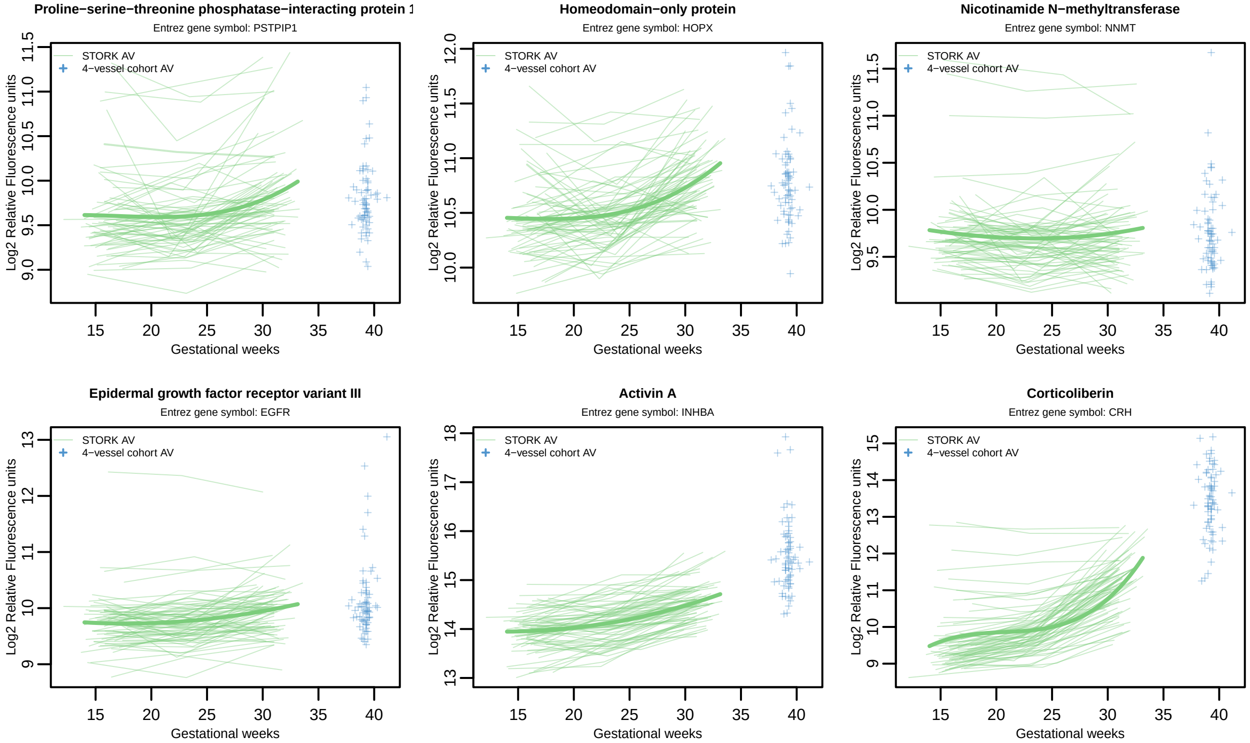
Proline−serine−threonine phosphatase−interacting protein 1
Entrez gene symbol: PSTPIP1
Homeodomain−only protein
Entrez gene symbol: HOPX
Nicotinamide N−methyltransferase
Entrez gene symbol: NNMT
Epidermal growth factor receptor variant III
Entrez gene symbol: EGFR
Activin A
Entrez gene symbol: INHBA
Corticoliberin
Entrez gene symbol: CRH
STORK AV
4−vessel cohort AV
Log2 Relative Fluorescence units
Gestational weeks
15
20
25
30
35
40

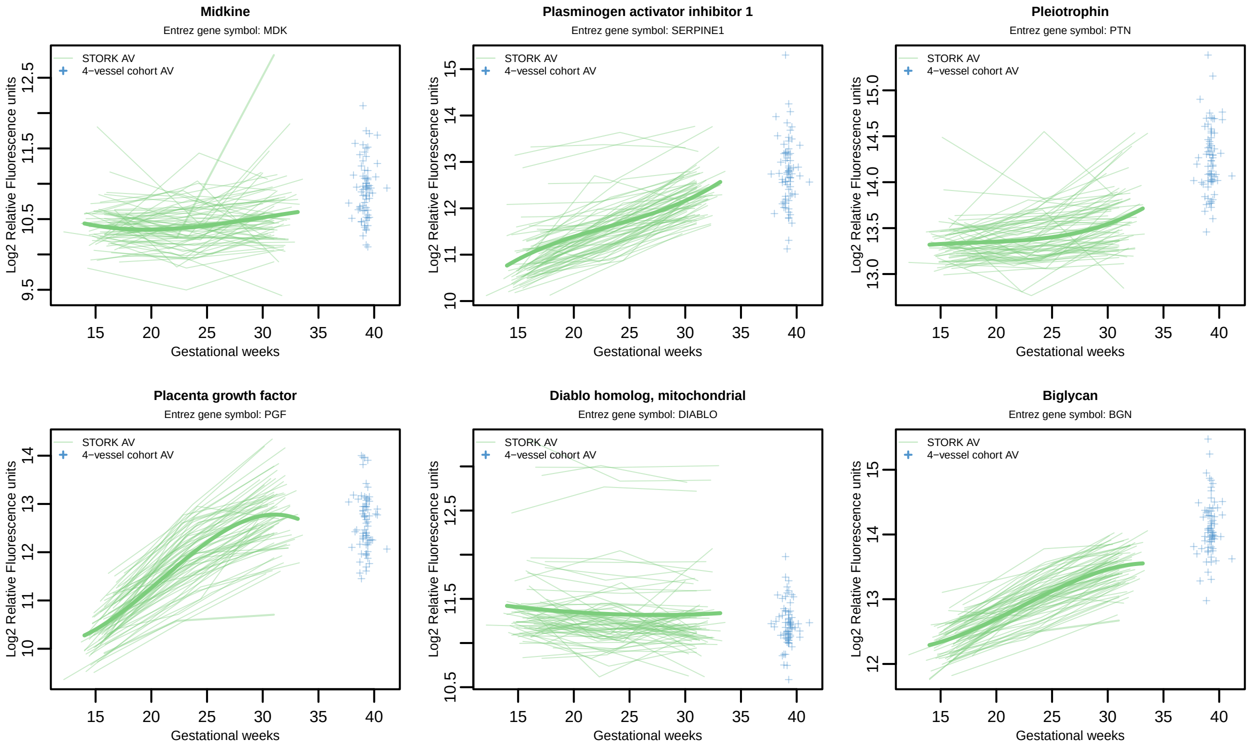

Midkine
Entrez gene symbol: MDK
Plasminogen activator inhibitor 1
Entrez gene symbol: SERPINE1
Pleiotrophin
Entrez gene symbol: PTN
Placenta growth factor
Entrez gene symbol: PGF
Diablo homolog, mitochondrial
Entrez gene symbol: DIABLO
Biglycan
Entrez gene symbol: BGN
STORK AV
4−vessel cohort AV
Log2 Relative Fluorescence units
Gestational weeks

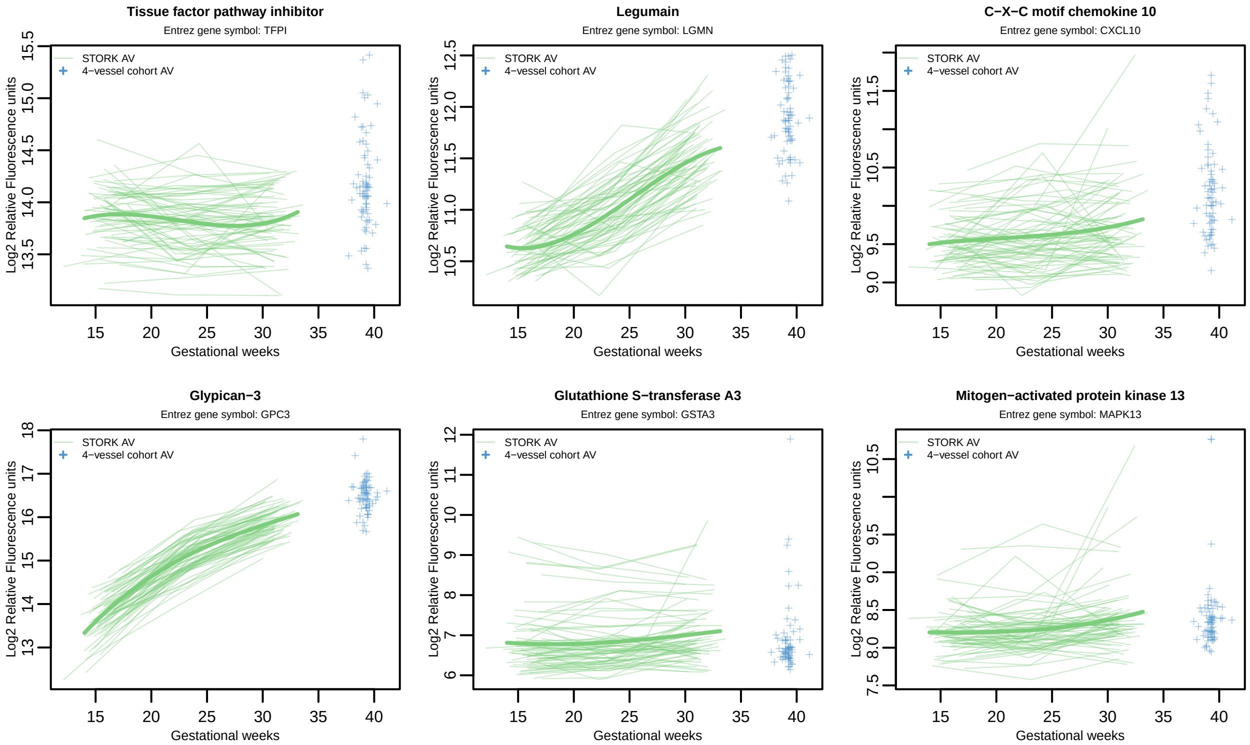

Tissue factor pathway inhibitor
Entrez gene symbol: TFPI
Legumain
Entrez gene symbol: LGMN
C−X−C motif chemokine 10
Entrez gene symbol: CXCL10
Glypican−3
Entrez gene symbol: GPC3
Glutathione S−transferase A3
Entrez gene symbol: GSTA3
Mitogen−activated protein kinase 13
Entrez gene symbol: MAPK13
STORK AV
4−vessel cohort AV
Log2 Relative Fluorescence units
Gestational weeks
15
20
25
30
35
40
13.5
14.0
14.5
15.0
15.5
10.5
11.0
11.5
12.0
12.5
9.0
9.5
10.5
11.5
13
14
15
16
17
18
6
7
8
9
10
11
12
7.5
8.0
8.5
9.0
9.5
10.5

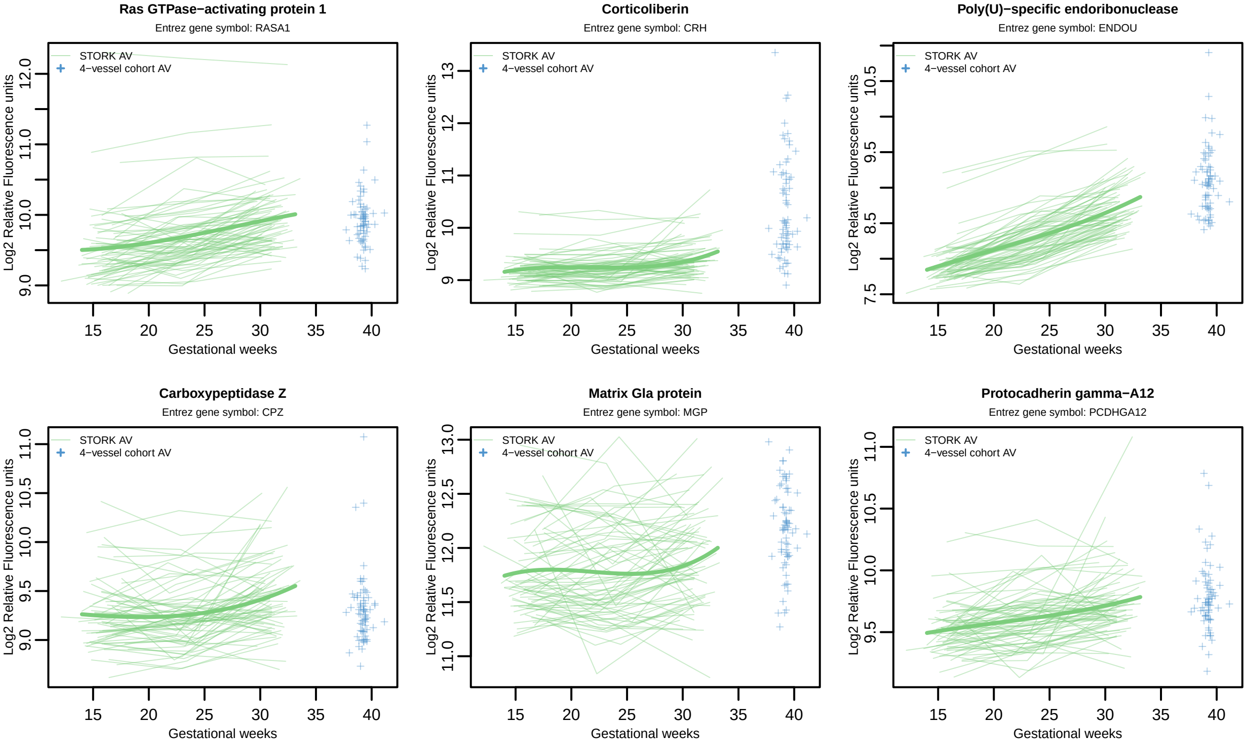
Ras GTPase−activating protein 1
Entrez gene symbol: RASA1
Corticoliberin
Entrez gene symbol: CRH
Poly(U)−specific endoribonuclease
Entrez gene symbol: ENDOU
Carboxypeptidase Z
Entrez gene symbol: CPZ
Matrix Gla protein
Entrez gene symbol: MGP
Protocadherin gamma−A12
Entrez gene symbol: PCDHGA12
STORK AV
4−vessel cohort AV
Log2 Relative Fluorescence units
Gestational weeks
15
20
25
30
35
40

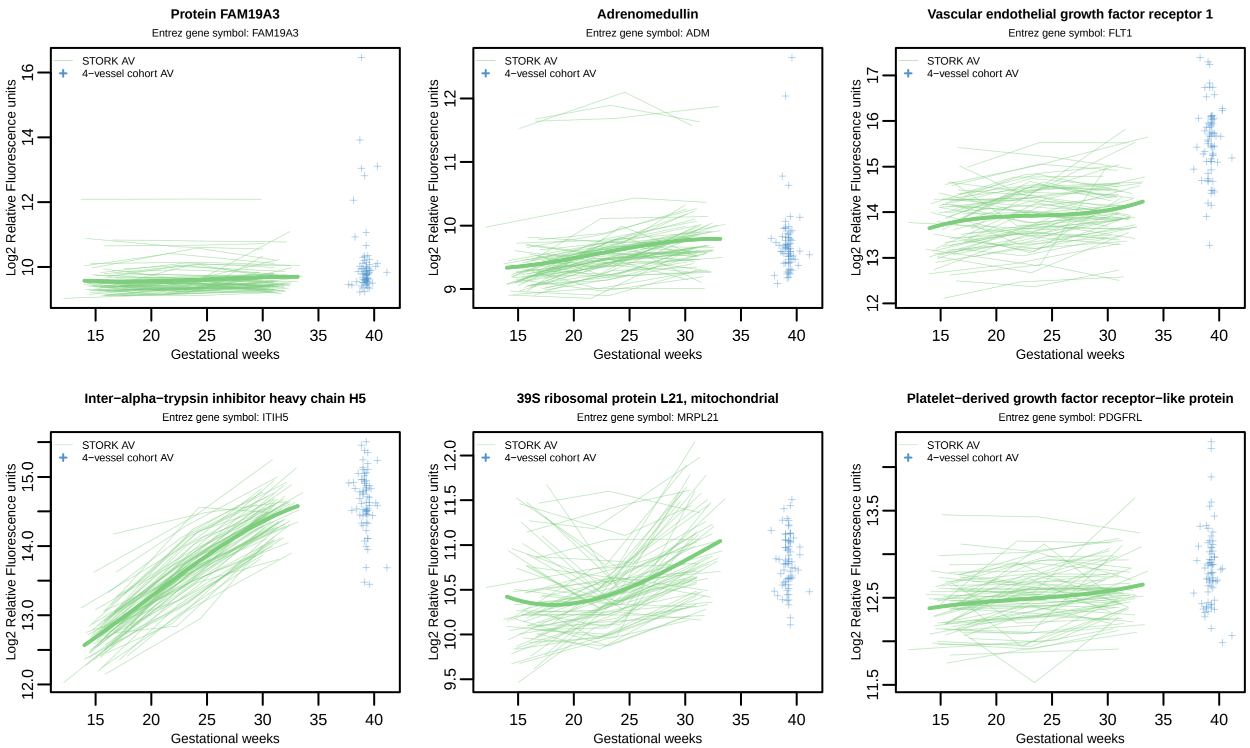

Protein FAM19A3
Entrez gene symbol: FAM19A3
Adrenomedullin
Entrez gene symbol: ADM
Vascular endothelial growth factor receptor 1
Entrez gene symbol: FLT1
Inter−alpha−trypsin inhibitor heavy chain H5
Entrez gene symbol: ITIH5
39S ribosomal protein L21, mitochondrial
Entrez gene symbol: MRPL21
Platelet−derived growth factor receptor−like protein
Entrez gene symbol: PDGFRL
STORK AV
4−vessel cohort AV
Log2 Relative Fluorescence units
Gestational weeks

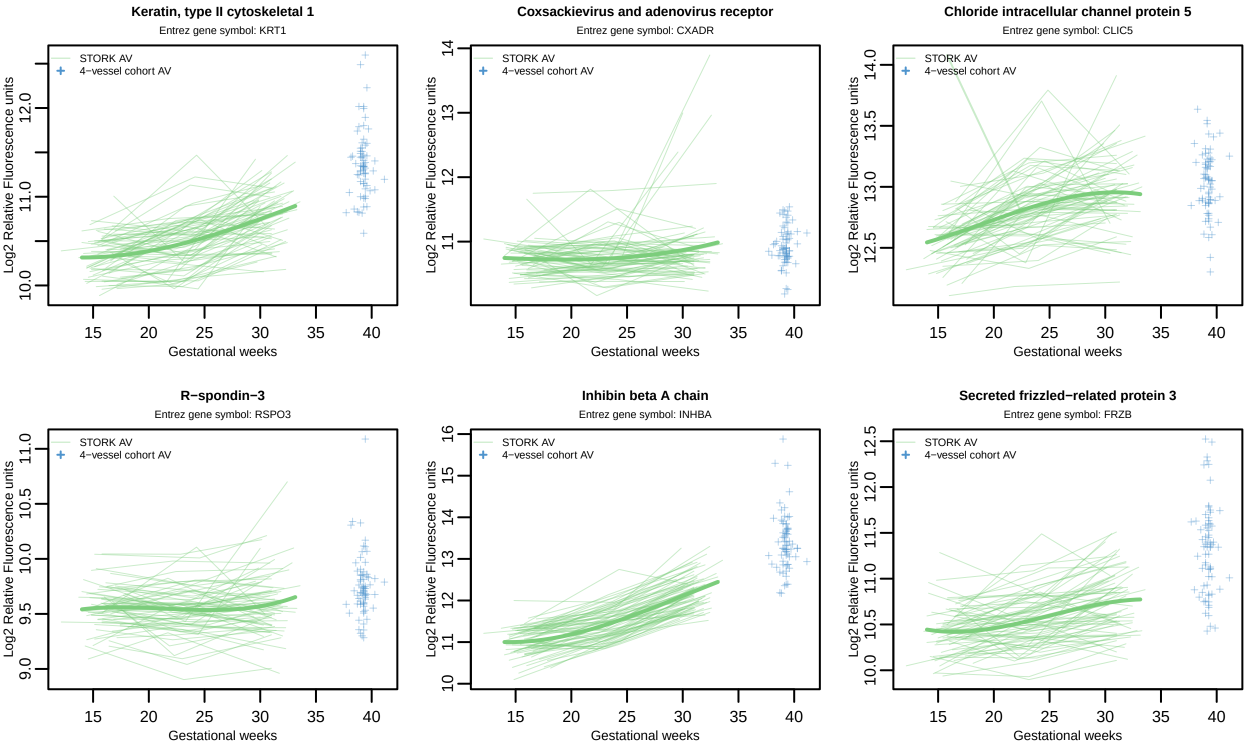

Keratin, type II cytoskeletal 1
Entrez gene symbol: KRT1
STORK AV
4−vessel cohort AV
Log2 Relative Fluorescence units
Gestational weeks
Coxsackievirus and adenovirus receptor
Entrez gene symbol: CXADR
STORK AV
4−vessel cohort AV
Log2 Relative Fluorescence units
Gestational weeks
Chloride intracellular channel protein 5
Entrez gene symbol: CLIC5
STORK AV
4−vessel cohort AV
Log2 Relative Fluorescence units
Gestational weeks
R−spondin−3
Entrez gene symbol: RSPO3
STORK AV
4−vessel cohort AV
Log2 Relative Fluorescence units
Gestational weeks
Inhibin beta A chain
Entrez gene symbol: INHBA
STORK AV
4−vessel cohort AV
Log2 Relative Fluorescence units
Gestational weeks
Secreted frizzled−related protein 3
Entrez gene symbol: FRZB
STORK AV
4−vessel cohort AV
Log2 Relative Fluorescence units
Gestational weeks

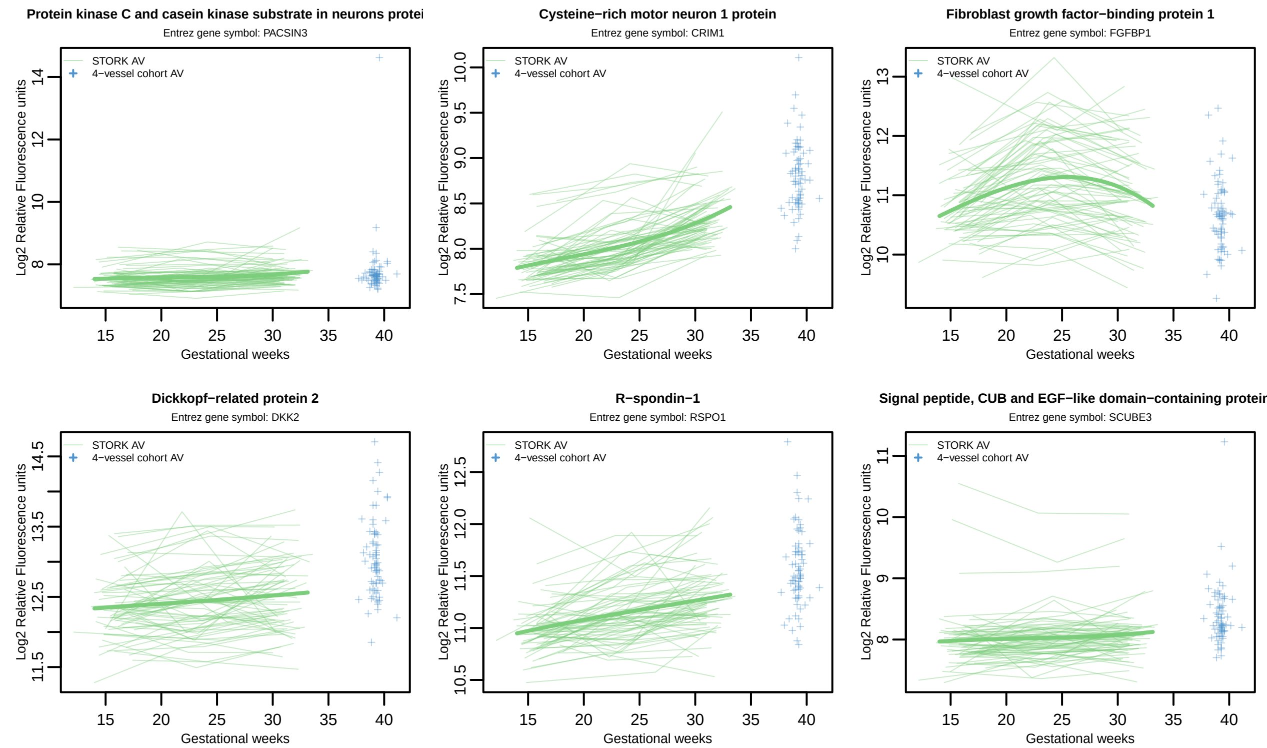
Protein kinase C and casein kinase substrate in neurons protei
Entrez gene symbol: PACSIN3
Cysteine−rich motor neuron 1 protein
Entrez gene symbol: CRIM1
Fibroblast growth factor−binding protein 1
Entrez gene symbol: FGFBP1
Dickkopf−related protein 2
Entrez gene symbol: DKK2
R−spondin−1
Entrez gene symbol: RSPO1
Signal peptide, CUB and EGF−like domain−containing protein
Entrez gene symbol: SCUBE3
STORK AV
4−vessel cohort AV
Log2 Relative Fluorescence units
Gestational weeks
15
20
25
30
35
40

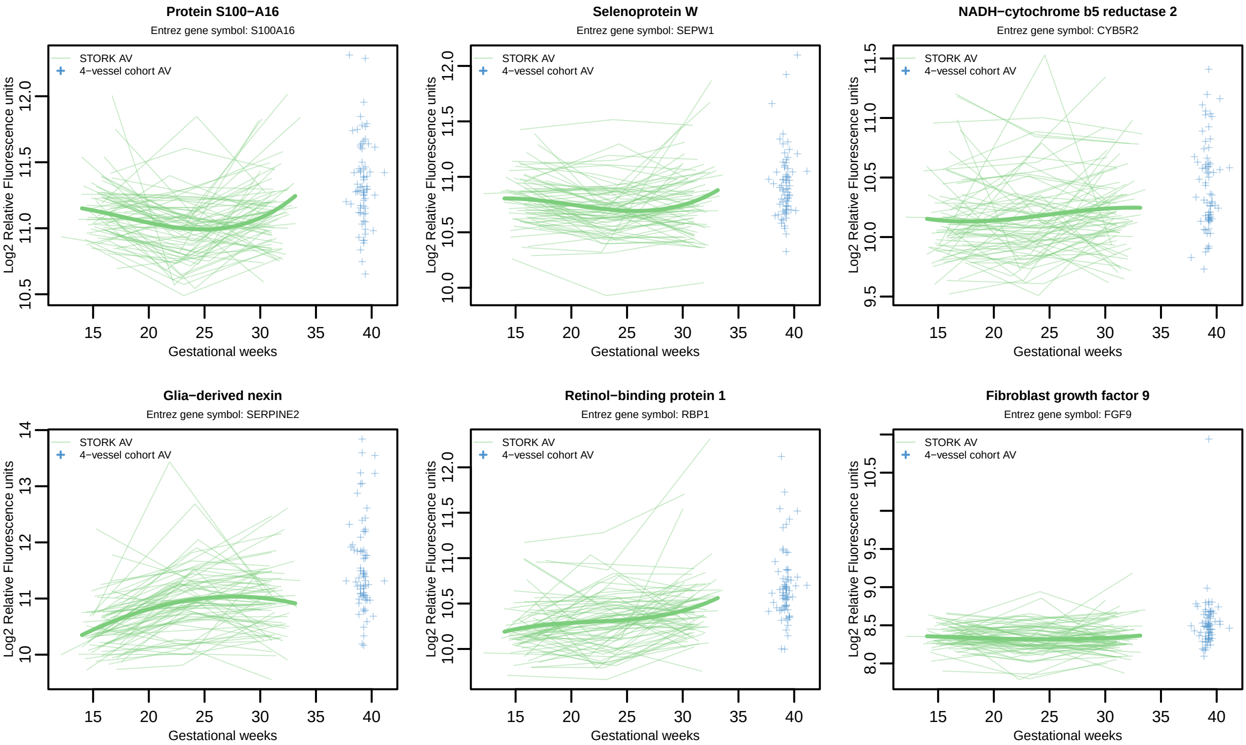
Protein S100−A16
Entrez gene symbol: S100A16
Selenoprotein W
Entrez gene symbol: SEPW1
NADH−cytochrome b5 reductase 2
Entrez gene symbol: CYB5R2
Glia−derived nexin
Entrez gene symbol: SERPINE2
Retinol−binding protein 1
Entrez gene symbol: RBP1
Fibroblast growth factor 9
Entrez gene symbol: FGF9
STORK AV
4−vessel cohort AV
Log2 Relative Fluorescence units
Gestational weeks
15
20
25
30
35
40

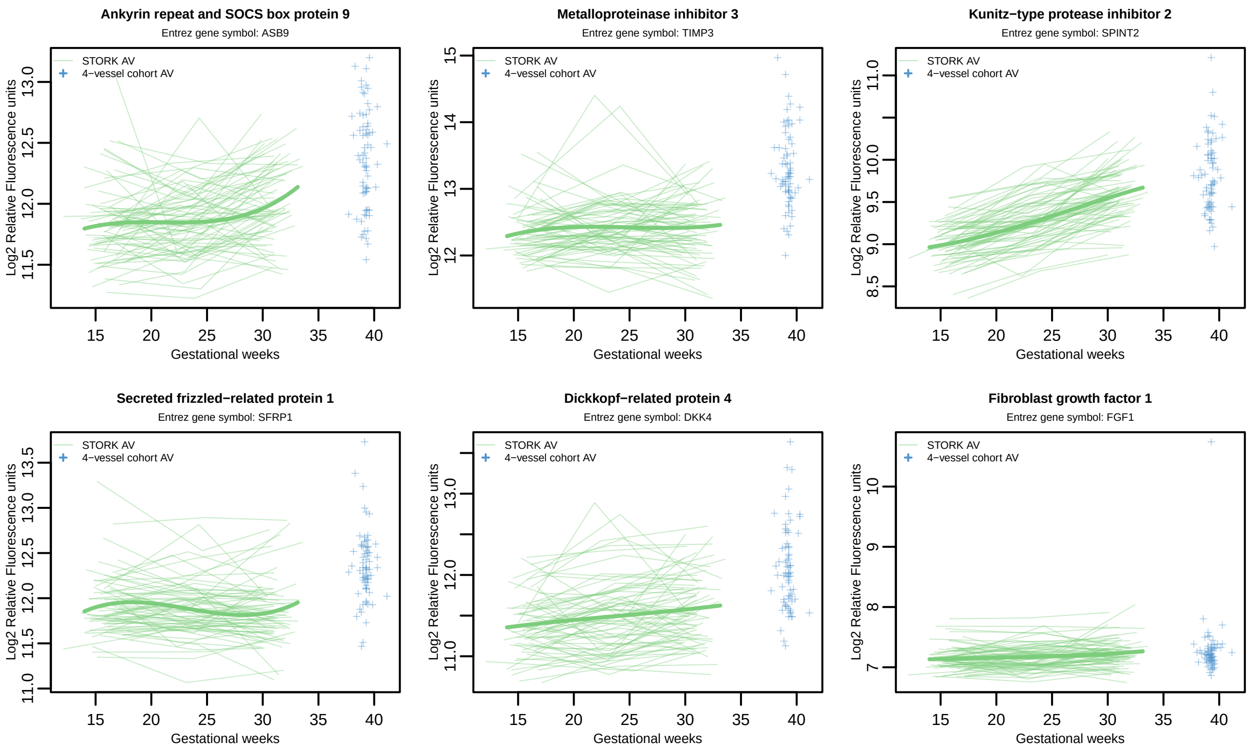

Ankyrin repeat and SOCS box protein 9
Entrez gene symbol: ASB9
Metalloproteinase inhibitor 3
Entrez gene symbol: TIMP3
Kunitz−type protease inhibitor 2
Entrez gene symbol: SPINT2
Secreted frizzled−related protein 1
Entrez gene symbol: SFRP1
Dickkopf−related protein 4
Entrez gene symbol: DKK4
Fibroblast growth factor 1
Entrez gene symbol: FGF1
STORK AV
4−vessel cohort AV
Log2 Relative Fluorescence units
Gestational weeks
15
20
25
30
35
40

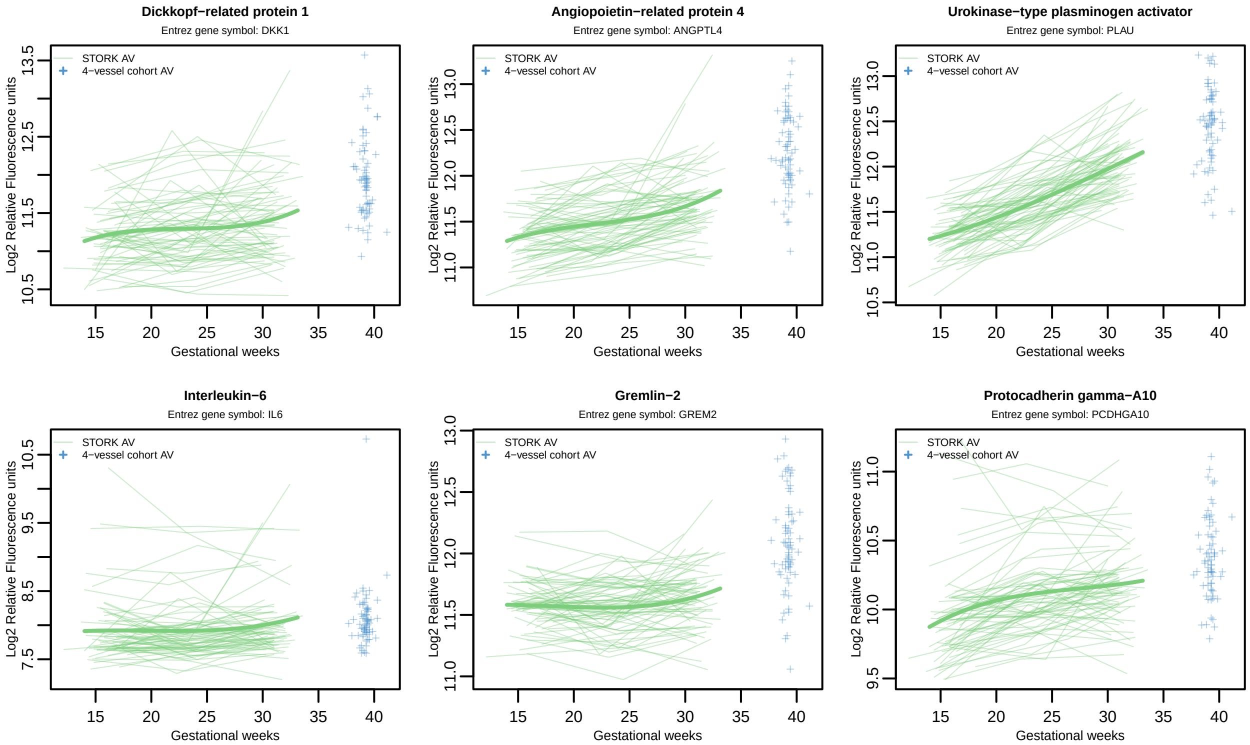
Dickkopf−related protein 1
Entrez gene symbol: DKK1
STORK AV
4−vessel cohort AV
Log2 Relative Fluorescence units
10.5
11.5
12.5
13.5
15
20
25
30
35
40
Gestational weeks
Angiopoietin−related protein 4
Entrez gene symbol: ANGPTL4
STORK AV
4−vessel cohort AV
Log2 Relative Fluorescence units
11.0
11.5
12.0
12.5
13.0
15
20
25
30
35
40
Gestational weeks
Urokinase−type plasminogen activator
Entrez gene symbol: PLAU
STORK AV
4−vessel cohort AV
Log2 Relative Fluorescence units
10.5
11.0
11.5
12.0
12.5
13.0
15
20
25
30
35
40
Gestational weeks
Interleukin−6
Entrez gene symbol: IL6
STORK AV
4−vessel cohort AV
Log2 Relative Fluorescence units
7.5
8.5
9.5
10.5
15
20
25
30
35
40
Gestational weeks
Gremlin−2
Entrez gene symbol: GREM2
STORK AV
4−vessel cohort AV
Log2 Relative Fluorescence units
11.0
11.5
12.0
12.5
13.0
15
20
25
30
35
40
Gestational weeks
Protocadherin gamma−A10
Entrez gene symbol: PCDHGA10
STORK AV
4−vessel cohort AV
Log2 Relative Fluorescence units
9.5
10.0
10.5
11.0
15
20
25
30
35
40
Gestational weeks

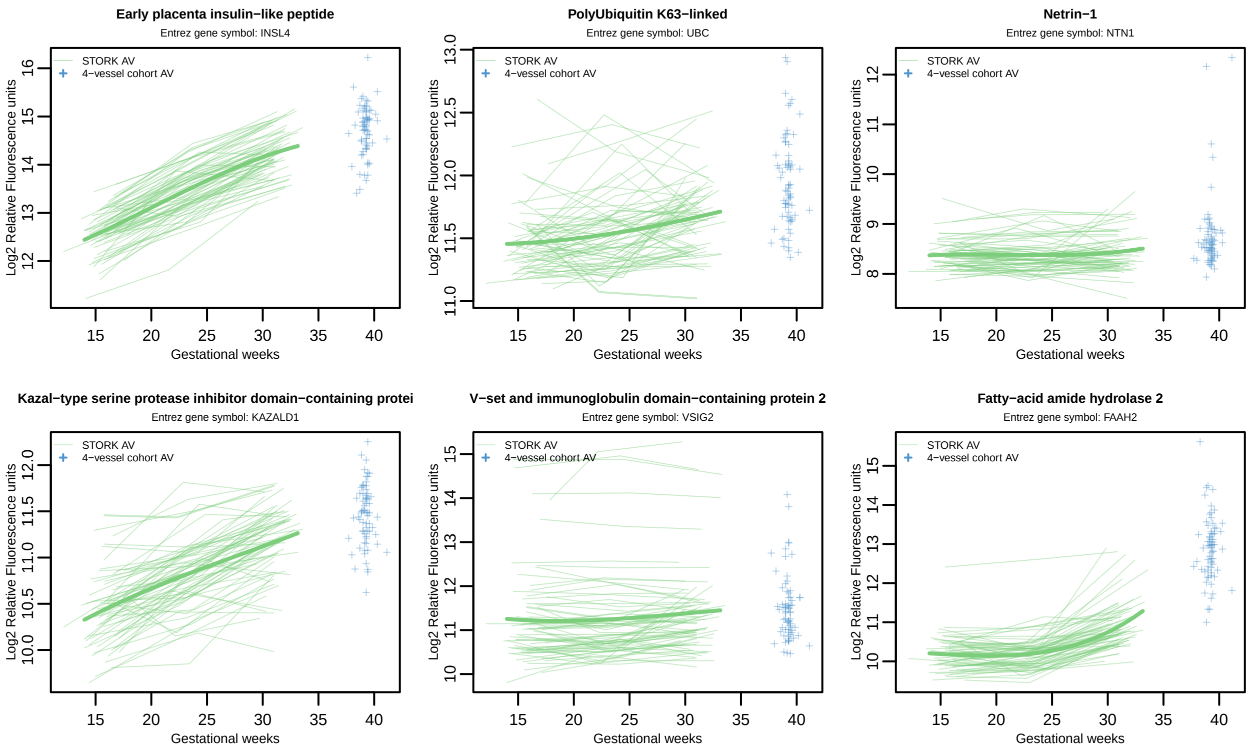
Early placenta insulin−like peptide
Entrez gene symbol: INSL4
PolyUbiquitin K63−linked
Entrez gene symbol: UBC
Netrin−1
Entrez gene symbol: NTN1
Kazal−type serine protease inhibitor domain−containing protei
Entrez gene symbol: KAZALD1
V−set and immunoglobulin domain−containing protein 2
Entrez gene symbol: VSIG2
Fatty−acid amide hydrolase 2
Entrez gene symbol: FAAH2
STORK AV
4−vessel cohort AV
Log2 Relative Fluorescence units
Gestational weeks
15
20
25
30
35
40

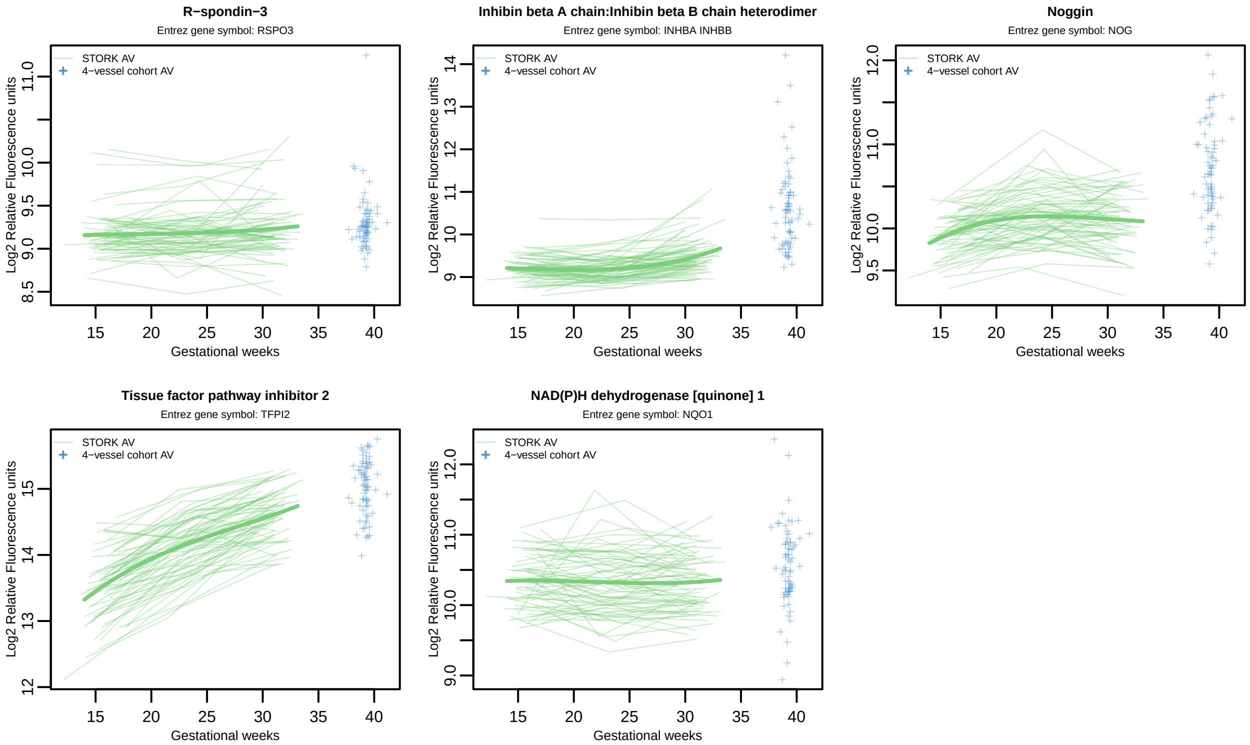
R−spondin−3
Entrez gene symbol: RSPO3
Inhibin beta A chain:Inhibin beta B chain heterodimer
Entrez gene symbol: INHBA INHBB
Noggin
Entrez gene symbol: NOG
Tissue factor pathway inhibitor 2
Entrez gene symbol: TFPI2
NAD(P)H dehydrogenase [quinone] 1
Entrez gene symbol: NQO1
STORK AV
4−vessel cohort AV
Log2 Relative Fluorescence units
Gestational weeks
15
20
25
30
35
40
